# A CDK4/6 inhibitor-armed oncolytic adenovirus reverses T cell exhaustion through the Rb-p65-CCL5 pathway and potentiates the antitumor activity of anti-PD-1 or CAR-T therapy in colorectal cancer

**DOI:** 10.3389/fimmu.2026.1839684

**Published:** 2026-06-15

**Authors:** Dan Zhou, Beibei Ran, Lingkai Kong, Yan Liu, Lingjun Xiao, Xiangmei Chen, Wencui Liu, Xiao Li, Jing Zhang, Jiahui Zhang, Hao Wu, Guang Zhang, Xiaosong Gu, Wenjie Zhang, Junhua Wu, Chunping Jiang

**Affiliations:** 1 State Key Laboratory of Pharmaceutical Biotechnology, Division of Hepatobiliary and Transplantation Surgery, Department of General Surgery Nanjing Drum Tower Hospital, Nanjing Drum Tower Hospital, the Affiliated Hospital of Medical School, Medical School, Nanjing University, Nanjing, China; 2 Jinan Microecological Biomedicine Shandong Laboratory, Building 1, Jinan Medical and Health Science and Technology Innovation Industrial Park, Jinan, Shandong, China; 3 Department of Hepatobiliary and Pancreatic Surgery, The Second Affiliated Hospital of Fujian Medical University, Quanzhou, Fujian, China; 4 Regional University–Industry Technology Transfer Center for Biopharmaceuticals (Nanjing, Jiangsu), Nanjing, China; 5 “Nanjing University-Gulou” Joint Laboratory of AI and Healthcare BigData, National Institute of Healthcare Data Science at Nanjing University, School of Life Sciences, Jiangsu Key Laboratory of Molecular Medicine, Nanjing University, Nanjing, China; 6 Renhuai People’s Hospital, Renhuai, Guizhou, China

**Keywords:** CD8, colorectal cancer, immunity, T cell, tumor

## Abstract

**Introduction:**

The efficacy of oncolytic adenoviruses in colorectal cancer models is constrained by a treatment-induced limitation: the high-dose, repetitive administration required for sustained oncolysis promotes chronic antigen exposure and tumor microenvironmental stress, driving CD8+ T cells into a state of exhaustion.

**Methods:**

To mitigate this, we constructed an oncolytic adenovirus, ADV-PTD4-D3, engineered for intratumoral expression of a peptide inhibitor of CDK4/6. This local strategy aims to retain immunomodulatory potential while minimizing systemic exposure. In syngeneic murine models, ADV-PTD4-D3 demonstrated improved tumor control and the ability to induce robust, antigen-specific immunological memory, with its therapeutic effect being primarily dependent on CD8+ T cells. Notably, it also exhibited potent antitumor activity in a humanized xenograft model and showed no evidence of significant off-target toxicity in immunocompetent hosts.

**Results:**

The mechanism involves a signaling axis where viral-mediated CDK4/6 inhibition reduces retinoblastoma (Rb) protein phosphorylation. This decrease relieves Rb-mediated sequestration of the NF-kB p65 subunit, allowing p65 nuclear translocation and transcriptional upregulation of the T-cell chemoattractant CCL5, a factor linked to favorable patient prognosis. Thus, ADV-PTD4-D3 promotes a T-cell-inflamed microenvironment by providing a sustained chemotactic signal CCL5 for CD8+ T cell recruitment. Furthermore, this treatment strategy successfully reverses the functional exhaustion of infiltrating CD8+ T cells, thereby addressing two major barriers to effective therapy: inadequate infiltration and functional exhaustion. By modifying the tumor microenvironment in this way, the armed virus addresses two factors that limit T-cell-based immunotherapies: inadequate infiltration and functional exhaustion. Correspondingly, ADV-PTD4-D3 treatment improved the antitumor response to both PD-1 blockade and CAR-T cell therapy in combination studies.

**Discussion:**

These findings suggest that engineering oncolytic viruses to locally modulate pathways involved in T cell exhaustion represents a viable and translatable strategy for enhancing antitumor immunity.

## Introduction

Colorectal cancer (CRC) remains a formidable clinical challenge, particularly for patients with microsatellite stable (MSS) disease who derive limited benefit from immune checkpoint blockade due to an immunologically “cold” tumor microenvironment (TME) lacking tumor-infiltrating lymphocytes (1). While oncolytic viruses (OVs) hold promise for reigniting anti-tumor immunity in such tumors (2–4), their therapeutic efficacy in CRC is constrained by a paradoxical immunological outcome inherent to current administration strategies (5–9).

To overcome this impasse, innovative strategies capable of decoupling potent oncolysis from the induction of T cell dysfunction are urgently required. Recently, cyclin-dependent kinase 4/6 (CDK4/6) inhibitors have garnered attention not only for their canonical role in inducing tumor cell cycle arrest but also as potent immunomodulatory agents. Beyond their direct anti-proliferative effects, CDK4/6 inhibition can enhance tumor antigen presentation and critically, promote the formation of durable T cell memory, thereby improving the quality of the anti-tumor immune response (10). Motivated by this rationale, we engineered a novel oncolytic adenovirus (11), designated ADV-PTD4-D3, which is armed with a genetic payload encoding a peptide inhibitor of CDK4/6 for specific, localized expression within the tumor. This design represents a strategic departure from systemic CDK4/6 inhibitor administration, which is frequently associated with dose-limiting off-target toxicities such as myelosuppression (8, 9, 12). By functioning as an *in situ* therapeutic factory, ADV-PTD4-D3 aims to achieve high intratumoral concentrations of the immunomodulator to disrupt tumor progression and modulate the local immune landscape, while ostensibly sparing systemic tissues. We hypothesized that this localized approach could address the twin fundamental barriers that limit the efficacy of T cell-directed therapies like PD-1 blockade and CAR-T cells in solid tumors: namely, inadequate T cell infiltration into the tumor core and the rapid functional exhaustion of those T cells that do manage to infiltrate (13–17).

In this study, we identify that the repetitive high-dose administration required to sustain viral load embodies a critical trade-off: while it significantly augments the absolute number of intratumoral CD8+ T cells (18), the resulting chronic antigen exposure, compounded by metabolic stress and inflammatory cytokine storm in the TME, mimics the conditions of chronic viral infection (19–21). This environment rapidly drives these cells into a state of profound exhaustion, characterized by the upregulation of inhibitory receptors and loss of effector function (14), engendering a self-limiting immune response, high in lymphocyte abundance but low in functional quality, that ultimately restricts durable tumor control.Furthermore, we elucidate the mechanism by which ADV-PTD4-D3 achieves this immunoremodeling, uncovering a novel signaling axis that bridges cell cycle regulation to innate inflammatory signaling. While the retinoblastoma (Rb) protein is best known for its role in regulating the E2F transcription factors to control cell cycle progression, emerging evidence indicates that its phosphorylated form (p-Rb) can exert non-canonical functions. Notably, p-Rb has been shown to physically sequester the NF-κB p65 subunit in the cytoplasm, thereby repressing the transcription of various immune-related genes (22). Here, we demonstrate that the CDK4/6 inhibitory activity of ADV-PTD4-D3 effectively blocks Rb phosphorylation in tumor cells. This dephosphorylation event relieves the inhibitory sequestration of p65 by Rb, facilitating p65 nuclear translocation. Once in the nucleus, p65 binds to and transactivates the promoter of the gene encoding CCL5, a potent T cell chemoattractant. Thus, ADV-PTD4-D3 amplifies the initial immunogenic cell death (ICD) signals elicited by viral oncolysis with a robust, sustained chemokine cue. This orchestrated response not only drives the targeted recruitment of CD8+ T cells but, more importantly, alters the TME to prevent their descent into an exhausted state. Furthermore, by establishing such a revitalized and T cell-inflamed microenvironment, this strategy creates a robust synergistic foundation for combination regimens. We demonstrate that ADV-PTD4-D3 significantly potentiates the therapeutic efficacy of both PD-1 immune checkpoint blockade and CAR-T cell therapy in models of refractory CRC.

## Materials and methods

### Construction of recombinant adenovirus

The adenovirus shuttle plasmid encoding the PTD4-D3 domain was obtained from Sino Biological. To facilitate the secretion and immunodetection of the peptide, an IL-2 signal peptide sequence (MYRMQLLSCIALSLALVTNS) was incorporated upstream (N-terminus). Furthermore, an IgG1 Fc linker and an HA epitope tag were fused downstream (C-terminus) of the coding sequence. The resulting pENTR entry plasmid was recombined with the pAd-DEST human adenovirus serotype 5 (Ad5) backbone vector to generate the recombinant adenoviral construct. Following linearization with the restriction enzyme *Pac*I, the construct was transfected into HEK-293T cells for viral rescue and amplification. Recombinant viral particles were purified via sucrose gradient ultracentrifugation, and viral titers were determined using the Adeno-X Rapid Titer Kit according to the manufacturer’s instructions.

### Cell lines

Human embryonic kidney cells (HEK-293T), human colorectal carcinoma cells (HCT116), and murine colorectal carcinoma cells (CT26) were obtained from the American Type Culture Collection (ATCC). The murine colorectal carcinoma cell line MC38 was acquired from the National Cancer Institute (NCI). All cell lines were authenticated by Short Tandem Repeat (STR) profiling and tested negative for mycoplasma contamination. WT murine CRC cells are naturally resistant to Ad5 infection due to low CAR expression. Therefore, CAR-expressing engineered lines were utilized to ensure efficient viral entry and replication across all models.MC38 and CT26 cells were engineered to stably express the Coxsackie and Adenovirus Receptor (CAR, encoded by the *Cxadr* gene), thereby rendering them permissive to adenovirus serotype 5 (Ad5)-based oncolytic viruses (23). For CAR-T studies, MC38-CAR cells were further engineered to express human CD19 (MC38-CAR-hCD19). All cell lines were maintained in Dulbecco’s Modified Eagle Medium (DMEM) supplemented with 10% fetal bovine serum (FBS) and cultured at 37 °C in a humidified incubator with 5% CO_2_. Engineered stable cell lines were maintained under appropriate antibiotic selection pressure. For all experiments reported in this study (both *in vitro* and *in vivo*), MC38 and CT26 refer specifically to the engineered MC38-CAR and CT26-CAR cell lines, respectively, to ensure consistent and efficient infection by our human Ad5-based oncolytic adenovirus vectors.

### Animal models and *in vivo* procedures

#### Mice and ethics statement

Female C57BL/6 and BALB/c mice (4–8 weeks old) were purchased from the Model Animal Research Center of Nanjing University (Nanjing, China). Immunodeficient NCG mice were obtained from Cyagen Biosciences (USA). All animal experiments were conducted in strict accordance with the protocols approved by the Institutional Animal Care and Use Committee (IACUC) of the Medical School of Nanjing University.

#### Subcutaneous tumor models and viral treatment

To establish syngeneic subcutaneous tumor models, MC38-CAR or CT26-CAR cells (1 × 10^6^ cells suspended in 100 μL PBS) in the exponential growth phase were harvested and inoculated subcutaneously into the right flank of the mice. When tumors reached an average volume of 50–100 mm³, mice were randomized into treatment groups. Intratumoral (i.t.) injections of PBS (100μL) or the indicated adenoviruses were administered every two days for a total of three doses. Tumor dimensions were measured every two days using digital calipers, and tumor volume was calculated using the formula: V = 0.5 × length × width². Mice were euthanized when tumor volume exceeded the humane endpoint of 1, 500 mm³. To prevent leakage during intratumoral (i.t.) administration, 100 µL of virus or PBS was delivered slowly over 10–15 seconds using a 30-gauge needle. Taking advantage of the relatively high compliance and elasticity of the CRC tumor stroma, a multi-point, fan-shaped injection technique was employed, and the needle was retained within the tumor for an additional 10 seconds post-injection to maximize viral retention.

Once tumors reached a mean volume of 50–100 mm³, mice were randomized into treatment groups. We defined Day 1 as the first day of viral administration. Mice received intratumoral (i.t.) injections of PBS or the indicated adenoviruses on Day 1, Day 3, and Day 5, following an every-other-day dosing regimen. Tumor volumes were measured every two days throughout the study to monitor the kinetic response to the initial and subsequent viral doses. The first measurement was taken on the same day as the administration of the first dose.

#### Combination therapy regimens

Maraviroc Combination: To investigate the role of the CCL5/CCR5 axis, the CCR5 antagonist Maraviroc (Selleck Chemicals) was dissolved in 10% DMSO in PBS and administered intraperitoneally (i.p.) at a dose of 30 mg/kg daily. Maraviroc treatment was initiated concurrently with the first viral injection and continued until the experimental endpoint.

PD-1 Blockade: Anti-PD-1 antibody (clone RMP1–14 BioXCell USA) was administered intraperitoneally (200 μg/mouse) every three days for three doses, with the initial dose given 24 hours prior to the first viral injection.

CAR-T Therapy: C57BL/6 mice bearing MC38-CAR-hCD19 tumors were treated with CAR-T cells (1× 10^6^ cells/mouse, i.v.) every three days for three doses. Notably, the CAR-T regimen was initiated one day before the start of viral treatment to evaluate the combination effect.

#### Humanized CDX model

To establish the humanized cell-derived xenograft (CDX) model, HCT116 cells were implanted subcutaneously into NCG mice. On the following day, human peripheral blood mononuclear cells (PBMCs; 3× 10^6^ cells/mouse), isolated from healthy donor blood, were injected intravenously to reconstitute the human immune system. Viral treatment was initiated when tumor volumes reached 50–100 mm³, following the same protocol described for syngeneic models. Human peripheral blood mononuclear cells (PBMCs) were isolated from blood samples of healthy donors. The study protocols involving human specimens were conducted in strict accordance with the Declaration of Helsinki and were approved by the Ethics Committee of Nanjing Drum Tower Hospital, The Affiliated Hospital of Nanjing University Medical School. Written informed consent was obtained from all donors prior to sample collection.

#### *In vivo* immune cell depletion

For CD8+ T cell depletion, C57BL/6 mice received intraperitoneal injections of 200μg anti-CD8α monoclonal antibody (BioXCell, Clone 2.43, USA) every two days for a total of three doses. To validate depletion efficiency, peripheral blood and tumor tissues were analyzed by flow cytometry 48 hours following the final antibody administration.

### Cell viability assay

CT26 and MC38 cells were seeded into 96-well plates at a density of $1\times 10^{4}$ cells per well. Following cell adherence, cultures were infected with the indicated adenoviruses at infection (MOI = 0, 1, 5, 10, 15, 20, 50). At 48 hours post-infection, 10 µL of Cell Counting Kit-8 (CCK-8) reagent (Beyotime Biotechnology, China) was added to each well, and the plates were incubated for an additional hour at 37 °C. Absorbance was measured at 450 nm using a microplate reader. Cell viability was calculated using the following formula: Cell Viability (%) = (A_treatment_ - A_blank_)/(A_control_ - A_blank_) × 100, where A represents the absorbance value.

### Crystal violet staining

Tumor cells were seeded into culture plates and infected with adenoviruses following the protocol described for the cell viability assay. At 48 hours post-infection (h.p.i.), the culture medium was aspirated, and cells were stained with crystal violet solution (Beyotime Biotechnology, China) for 5 minutes at room temperature. The staining solution was subsequently discarded, and the plates were rinsed five times with double-distilled water ( ${\text{ddH}}_{2}\text{O}$) prior to imaging.

### Viral transduction and replication kinetics

To evaluate viral transduction efficiency, CT26 and MC38 cells were seeded into 24-well plates at a density of $5\times 10^{4}$ cells per well. Cells were infected with EGFP-expressing adenoviruses at multiplicities of infection (MOIs) of 0.1, 1, and 2. EGFP expression was quantified via flow cytometry at 48 hours post-infection (h.p.i.). For the assessment of viral replication kinetics, cells were seeded in 24-well plates and infected with adenoviruses at MOIs of 0.01 and 0.1. Following a 2-hour adsorption period, the viral inoculum was removed, cells were washed, and fresh culture medium was added. Cells were harvested at defined time points (24, 36, 48, 72, and 96 h.p.i.) to quantify viral load.

### Enzyme-linked immunosorbent assay

Cell culture supernatants from treated tumor cells were collected and centrifuged at 1, 000xg for 20 minutes at 4 °C to remove cellular debris. The concentration of murine CCL5 was quantified using a commercial ELISA kit (Elabscience, China) in strict accordance with the manufacturer’s instructions.

### RNA extraction and quantitative real-time PCR

Total RNA was extracted from cells using TRIzol reagent (Vazyme, China). Subsequently, RNA was reverse-transcribed into cDNA using the HiScript III RT SuperMix for qPCR (Vazyme, China). Quantitative PCR was performed using ChamQ SYBR qPCR Master Mix (Vazyme, China) on an Applied QuantStudio™ 5 Real-Time PCR System (Thermo Fisher Scientific, USA). Relative mRNA expression levels were normalized to the internal control *GAPDH*. The specific primer sequences used in this study are listed in **Supplementary Table**.

### Western blotting

Tumor cells were seeded in 10-cm culture dishes and infected with ADV or ADV-PTD4-D3 at a multiplicity of different infection MOIs. At indicated time points (18 hours for signaling pathway analysis or 48 hours for protein expression validation), cells were lysed in RIPA lysis buffer (Beyotime, China) supplemented with protease and phosphatase inhibitors (NCM Biotech, China). Protein concentration was determined using an Enhanced BCA Protein Assay Kit (Beyotime, China). Equal amounts of protein were resolved by 8% SDS-PAGE and transferred onto PVDF membranes (Millipore, USA). Membranes were blocked with 5% non-fat milk in TBST for 1 hour at room temperature. Membranes were then probed with primary antibodies against HA, phospho-Rb (Santa Cruz S249/T252, USA), or β-actin (Santa Cruz, USA) overnight at 4 °C, followed by incubation with horseradish peroxidase (HRP)-conjugated anti-rabbit or anti-mouse IgG secondary antibodies for 1 hour at room temperature. Blot images were captured using a chemiluminescent detection system (BIO-RAD, ChemiDoc™ MP Imaging System).

### *In vivo* bio-distribution analysis

To assess the systemic distribution of the PTD4-D3 peptide, tumor tissues, serum, heart, and liver samples were harvested 48 hours after the final (third) viral injection from ADV-PTD4-D3-treated mice. These tissues were lysed, and the expression of the HA-tagged PTD4-D3 peptide was evaluated via Western blotting using the aforementioned procedure.

### Transwell chemotaxis assay

Chemotaxis assays were performed using 24-well Transwell inserts with a 5.0 μm pore size (Corning, USA).

Lower Chamber Preparation: Tumor cells were seeded into the lower chambers at a density of 2×10^5^cells/well. Following adherence, cells were infected with PBS (mock), ADV, or ADV-PTD4-D3 at an MOI of 10. Four hours post-infection, the culture medium was replaced with low-serum medium (containing 1% FBS), and cells were cultured for an additional 48 hours to allow for chemokine accumulation.

Upper Chamber Preparation: CD8+ T cells were isolated from murine spleens using magnetic bead separation and activated with anti-CD3/anti-CD28 antibodies for 48 hours to induce chemokine receptor expression. Activated T cells (2×10^5^) were resuspended in 100 μL of serum-free RPMI-1640 medium and loaded into the upper inserts.

Migration Analysis: The system was incubated at 37 °C in a 5% CO_2_ atmosphere for 4 hours. Cells that migrated to the lower chamber were collected, mixed with absolute counting beads (Invitrogen, USA), and quantified by flow cytometry.

### CCR5 blocking assay

To validate the role of the CCR5 axis, a blocking assay was performed using the specific CCR5 antagonist Maraviroc (MVC). A chemotactic gradient was established in the lower chambers using virus-infected tumor cells as described above. Prior to the migration assay, activated CD8+ T cells were harvested and pre-incubated with MVC (10 μM) for 1 hour at 37 °C. Pre-treated T cells were then loaded into the upper inserts in the continued presence of the drug or vehicle. After 4 hours of migration, the blocking efficiency was assessed by quantifying migrated cells via flow cytometry.

### Flow cytometry

Tumor tissues were minced into small pieces and digested in DMEM containing Collagenase IV (500 µg/mL) and DNase I (100 µg/mL) for 1 hour at 37 °C. The digested tissue was filtered to obtain single-cell suspensions. Dead cells were excluded by staining with a Fixable Viability Dye (BioLegend) prior to fixation. To minimize non-specific binding, cells were pre-incubated with an Fc receptor block (anti-mouse CD16/32, Clone 93, Cat101302, BioLegend) for 10 minutes at 4 °C. Surface staining was then performed with fluorophore-conjugated antibodies. For intracellular staining of cytokines and cytotoxic molecules, cells were subsequently fixed and permeabilized using a Cyto-Fast™ Fix/Perm Buffer Set (BioLegend) according to the manufacturer’s instructions, followed by staining with intracellular antibodies. Data acquisition was performed on a CytoFLEX S flow cytometer (Beckman Coulter), and data were analyzed using FlowJo v10 software. The following antibodies (all from BioLegend) were used: anti-mouse CD45-APC(Clone30-F11, Cat103112), CD4-PE/Cy7(Clone GK1.5, Cat100422), CD11b-FITC(CloneM1/70, Cat101206), CD49b-PE(CloneDX5, Cat108908), NK1.1-FITC(ClonePK136, Cat108706), F4/80-PE/Cy7(Clone BM8, Cat123114), CD86-PE(CloneGL-1, Cat105008), CD206-PerCP/Cy5.5(CloneC068C2, Cat141716), CD3-APC/Cy7(Clone 17A2, Cat100222), CD8-PerCP(Clone 53-6.7, Cat100732), CD62L-APC(Clone W18021D, Cat104412), CD44-PE(Clone IM7, Cat103007), CD45-FITC(Clone30-F11, Cat103108), CD11c-PE/Cy7(CloneN418, Cat117318), PD-1-PE/Cy7(Clone 29F.1A12, Cat135216), TIM-3-APC(Clone RMT3-23, Cat119738), IFN-γ-PE(Clone XMG1.2, Cat505808), Granzyme B-FITC (Clone QA16A02, Cat372206)and MHC II-PE(Clone M5/114.15.2, Cat107608)(BioLegend, China).

### Histological analysis (H&E staining)

Murine organ tissues were fixed in 4% (w/v) paraformaldehyde (Sigma-Aldrich, Germany) and embedded in paraffin blocks. Sections of 5 μm thickness were cut, deparaffinized in xylene, and rehydrated through a graded ethanol series. The sections were subsequently subjected to standard hematoxylin and eosin (H&E) staining for pathological evaluation.

### Chromatin immunoprecipitation-qPCR

ChIP assays were performed using the BeyoChIP™ ChIP Assay Kit (Beyotime, Cat. No. P2080S China) following the manufacturer’s instructions. Tumor cells were infected with PBS, ADV, or ADV-PTD4-D3 at a multiplicity of infection (MOI) of 20. At 48 hours post-infection, cells were cross-linked by adding formaldehyde directly to the culture medium to a final concentration of 1% for 10 minutes at room temperature. The cross-linking reaction was quenched by the addition of glycine to a final concentration of 0.125 M for 5 minutes.

Cells were harvested, lysed, and sonicated on ice to shear chromatin DNA into fragments ranging from 200 to 500 bp in length. Following centrifugation to remove insoluble debris, supernatants were incubated overnight at 4 °C with rotation in the presence of a ChIP-grade anti-p65 antibody (Proteintech, USA) or a normal IgG isotype control. Protein A+G magnetic beads were subsequently added and incubated for 2 hours at 4 °C to capture the antibody-chromatin complexes. The beads were collected using a magnetic separator and subjected to stringent washing sequentially with low-salt, high-salt, and LiCl immune complex wash buffers, followed by elution of the chromatin.

Cross-links were reversed by incubating the eluates in the presence of 0.2 M NaCl at 65 °C overnight. The samples were then digested with RNase A and Proteinase K to remove RNA and proteins, respectively. DNA was purified and analyzed by quantitative real-time PCR (qPCR) using SYBR Green Master Mix. Specific primers targeting the predicted NF-κB binding site within the *CCL5* promoter region were used (sequences are listed in **Supplementary Table**).

ChIP enrichment was quantified as fold enrichment relative to the IgG isotype control. The relative occupancy of p65 at the CCL5 promoter was calculated using the formula: Fold Enrichment = 2 ^ (Ct_IgG_ - Ct_IP_), where Ct_IgG_ and Ct_IP_ represent the Ct values of the IgG control and the anti-p65 immunoprecipitates, respectively. All ChIP-qPCR experiments were performed in three independent biological replicates.

### Immunohistochemistry

Tumor tissues were harvested 48 hours after the final (third) viral injection, fixed in 4% paraformaldehyde for 24 hours, and embedded in paraffin. Five-micrometer sections were deparaffinized and rehydrated for subsequent staining. Antigen retrieval was performed using citrate buffer (pH 6.0) at 95 °C for 20 minutes. After blocking with 5% BSA, sections were incubated with primary antibody against Cleaved Caspase-3 overnight at 4 °C, followed by incubation with an HRP-conjugated secondary antibody. Signals were visualized using a 3, 3’-diaminobenzidine (DAB) substrate kit, and nuclei were counterstained with hematoxylin. Positive cells were quantified using ImageJ software, with data expressed as the number of positive cells per high-power field (HPF).

### Hematological analysis

Peripheral blood samples were collected via orbital bleeding 48 hours after the final viral injection. Complete blood counts (CBC), including white blood cell (WBC) and neutrophil (Neu#) counts, were analyzed using an automated hematology analyzer to assess potential hematopoietic toxicity. All procedures were performed in strict accordance with the manufacturer’s instructions.

### Bioinformatics analysis

To evaluate the expression profile of CCL5 in colorectal cancer, we utilized the Gene Expression Profiling Interactive Analysis (GEPIA) web server (http://gepia.cancer-pku.cn/), which integrates RNA sequencing expression data from The Cancer Genome Atlas (TCGA). Differential expression of CCL5 was analyzed within the Colon Adenocarcinoma (COAD) cohort, comprising 275 tumor samples and 41 normal tissue samples. Gene expression data were normalized and presented on a log_2_ [Transcripts Per Million (TPM) + 1] scale. Statistical significance between the tumor and normal groups was determined using one-way ANOVA, with a P-value < 0.01 considered statistically significant.

The prognostic value of CCL5 in colorectal cancer was assessed using the Kaplan-Meier Plotter database (http://kmplot.com/analysis/), an online repository integrating gene expression and clinical data from GEO, EGA, and TCGA. Affymetrix probe ID 1555759_a_at was selected to represent CCL5 expression. Patients were stratified into high- and low-expression groups based on the “auto-select best cutoff” algorithm to maximize the distinction between survival cohorts. The association between CCL5 expression and overall survival (OS) was evaluated using the Kaplan-Meier method, and statistical significance was determined by the log-rank test. Hazard ratios (HR) with 95% confidence intervals (CI) were calculated using univariate Cox proportional hazards regression. A P-value < 0.05 was considered statistically significant.

The immunological landscape of CCL5 was evaluated using the TIMER3 web server (https://compbio.cn/timer3/). We performed a comprehensive analysis to correlate gene expression with CD8+ T cell infiltration levels across diverse cancer types. To ensure the robustness of the estimation, we employed a wide array of immune deconvolution algorithms provided by the platform, including TIMER, EPIC, MCP-counter, CIBERSORT, CIBERSORT-ABS, QUANTISEQ, XCELL, ABIS, ImmuCellAI, and TIDE. The association between gene expression and immune cell abundance was assessed using purity-adjusted partial Spearman’s correlation. In the resulting heatmap, the color scale indicates the partial correlation coefficient (Partial_Cor), and statistical significance (*P*<0.05) is denoted by solid squares, while non-significant results (*P*>0.05) are marked with a cross (×).

### Quantification and statistical analysis

The statistical significance of differences in multiple groups was analyzed using GraphPad Prism by one-way ANOVA. The survival of tumor-bearing mice was analyzed using the Kaplan–Meier method with the log rank test. Student’s t test or paired t test was used to compare two independent or matched groups. Data distribution was assumed to be normal, but this was not formally tested. Data are shown as the mean ± SD (NS, no significant differences; ∗*p* < 0.05, ∗∗*p* < 0.01, ∗∗∗*p* < 0.001, ∗∗∗∗*p* < 0.0001).

## Result

### Repeated administration of oncolytic adenovirus triggers antitumor immunity but concomitant CD8+ T cell exhaustion

**Figure 1:**
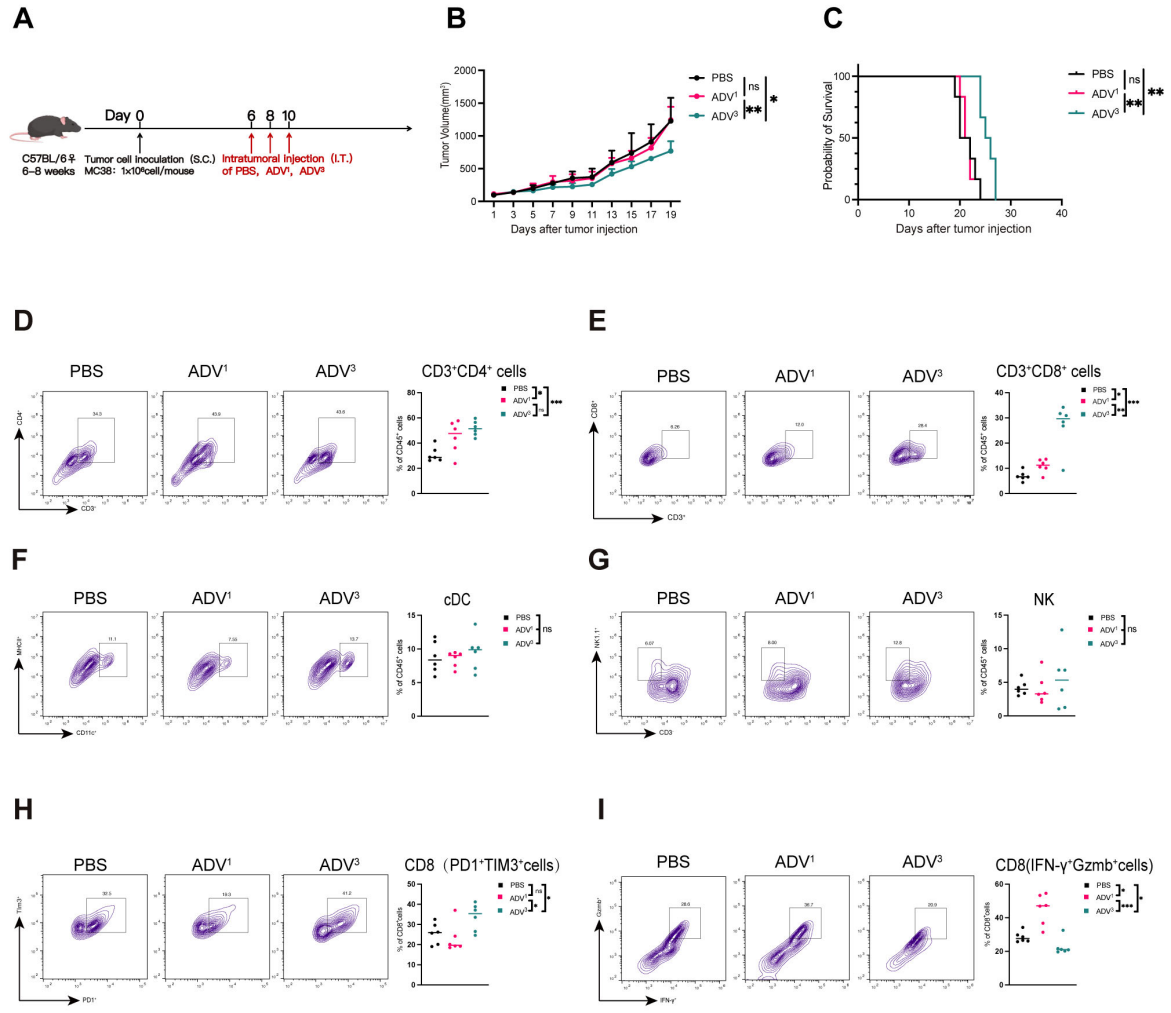
Anti-tumor efficacy and immune modulation of oncolytic adenovirus (ADV) at varying dosing regimens. **(A)** Schematic illustration of the experimental design and treatment timeline. MC38 were subcutaneously implanted into wild-type mice. Upon reaching a tumor volume of approximately 50–100 mm³, mice were randomized to receive either a single dose (ADV1) or three doses administered every other day (ADV3) of ADV (5 × 10^8^ PFU). **(B)** Tumor growth kinetics of tumor-bearing mice following treatment. **(C)** Kaplan-Meier survival analysis of tumor-bearing mice. **(D, E)** Quantification of adaptive T cell infiltration. The percentages of **(D)** CD3+CD4+ T cells and **(E)** CD3+CD8+ T cells within the tumor microenvironment were assessed by flow cytometry (n = 6 biological replicates). **(F, G)** Flow cytometric analysis of innate immune infiltration one day after the final treatment. The intratumoral percentages of **(F)** dendritic cells (DCs) and **(G)** natural killer (NK) cells were quantified (n = 6 biological replicates). **(H, I)** Assessment of CD8+ T cell exhaustion and effector function one day post-treatment. **(H)** The proportion of exhausted CD8+ T cells (PD-1+TIM-3+) and **(I)** functional effector CD8+ T cells (IFN-γ+Granzyme B+) were analyzed (n = 6 biological replicates). Data are presented as mean ± SD. NS, not significant; *P < 0.05, **P < 0.01, ***P < 0.001.

We initiated our study by assessing the therapeutic potential of a wild-type oncolytic adenovirus (ADV) in syngeneic murine models of colorectal cancer (**Figure 1A**). Intratumoral injection of a repeated ADV regimen (ADV3), but not a single dose (ADV1), elicited potent tumor suppression (**Figure 1B**) and significantly prolonged the survival of tumor-bearing mice compared to PBS controls (**Figure 1C**). Further investigation revealed that this antitumor effect was not solely due to direct oncolysis but relied substantially on remodeling the tumor immune microenvironment (TME). ADV treatment effectively breached immune tolerance, converting immunologically “cold” tumors into “hot” tumors, as evidenced by significant infiltration of CD3+CD4+ and CD3+CD8+ T cells (**Figures 1D, E**). In contrast, the infiltration levels of natural killer (NK) cells or dendritic cells (DCs) were not significantly altered (**Figures 1F, G**), indicating a T cell-dominated adaptive immune response.

However, this potent antitumor response was accompanied by a paradoxical induction of CD8+ T cell dysfunction. The high-dose ADV regimen successfully expanded the intratumoral effector T cell pool, yet the resulting chronic antigen exposure, driven by persistent viral replication and tumor lysis, precipitated a state of T cell overstimulation. Flow cytometric analysis confirmed that CD8+ T cells in the high-dose group exhibited significantly elevated co-expression of the exhaustion markers PD-1 and TIM-3 (**Figure 1H**). This phenotypic shift was coupled with a profound functional impairment, as evidenced by the substantial downregulation of the key effector cytokines IFN-γ and Granzyme B (**Figure 1I**). These data demonstrate that within the ADV-inflamed TME, infiltrating CD8+ T cells rapidly transition from an activated to an exhausted state.

Collectively, these findings establish that the efficacy of high-dose oncolytic virotherapy is intrinsically limited by a self-reinforcing inhibitory loop: the robust immunogenic cell death and T cell recruitment it instigates ultimately lead to iatrogenic T cell exhaustion. This inherent trade-off underscores the necessity for combinatorial strategies designed to preserve T cell fitness, which we pursued through the rational engineering of an armed oncolytic viral platform.

### Engineering and *in vitro* validation of ADV-PTD4-D3, an oncolytic adenovirus armed with a CDK4/6 inhibitory peptide

Cyclin-dependent kinases 4/6 (CDK4/6) serve as core regulators of the cell replication cycle, and their dysregulation is intimately linked to aberrant proliferation across various malignancies (24, 25). Pharmacological studies have demonstrated that CDK4/6 inhibitors exhibit significant therapeutic activity in multiple solid tumors (26, 27). Conventionally, the mechanism of action is attributed to the induction of G1 phase cell cycle arrest via the inhibition of Retinoblastoma (Rb) protein phosphorylation. However, accumulating evidence suggests that selective CDK4/6 inhibitors possess the potential to promote anti-tumor immune responses beyond mere cell cycle control (10). Based on this rationale, we engineered an oncolytic adenovirus to express a CDK4/6 inhibitory function. This strategy aims not only to potentiate apoptosis induction in tumor cells but also to resolve the CD8+ T cell exhaustion observed in colorectal cancer models following repetitive ADV administration, thereby remodeling a cytotoxic immune microenvironment. PTD4-D3 is a well-established peptide known to exert therapeutic and immunogenic effects in various tumor types (28).

**Figure 2:**
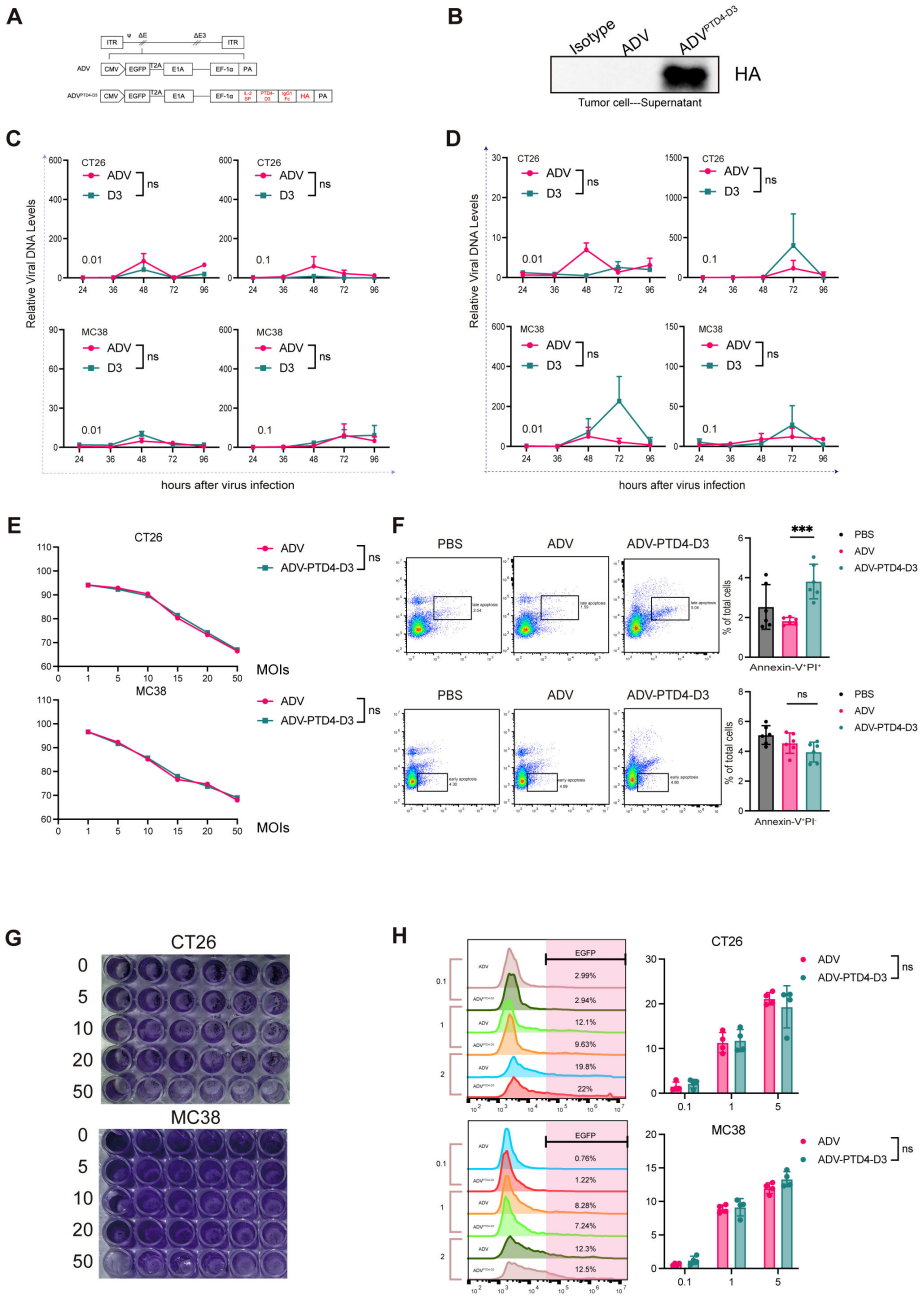
Construction and *in vitro* characterization of the recombinant oncolytic adenovirus ADV-PTD4-D3. **(A)** Schematic illustration of the ADV-PTD4-D3 genomic construct. Schematic representation of the ADV-PTD4-D3 genomic construct. The expression cassette includes a CMV promoter, an IL-2 signal peptide, the PTD4-D3 payload, and a C-terminal IgG1 Fc-HA tag. Detailed construction and functional rationale for these components are described in the Materials and Methods section. **(B)** Validation of PTD4-D3 peptide secretion. MC38 cells were infected with ADV or ADV-PTD4-D3 at a multiplicity of infection (MOI) of 2 for 48 hours. Protein secretion in the supernatant was confirmed via Western blotting. **(C, D)** Assessment of viral infectivity and replication kinetics. CT26 and MC38 cells were infected at the indicated MOIs, and viral gene expression was quantified by qPCR at 24, 36, 48, 72, and96 hours post-infection (h.p.i.). The decrease in viral load at later time points is attributed to cell lysis and detachment. **(E)** Evaluation of cell viability. CT26 and MC38 cells were infected with ADV or ADV-PTD4-D3 at indicated MOIs (0-50), and cell viability was assessed via CCK-8 assay at 48 h.p.i. **(F)** Analysis of virus-induced apoptosis. MC38 cells were infected with ADV or ADV-PTD4-D3 (MOI = 5) for 12 hours. Apoptotic populations were characterized by flow cytometry, with early apoptotic cells defined as Annexin-V+/PI- and late apoptotic cells as Annexin-V+/PI+ (n = 6 biological replicates). **(G)** Determination of oncolytic cytotoxicity. CT26 and MC38 cells were infected at the indicated MOIs for 48 hours, followed by visualization and quantification using crystal violet staining (n = 6 biological replicates). **(H)** Viral transduction efficiency. EGFP reporter expression in CT26 and MC38 cells infected with ADV or ADV-PTD4-D3 at various MOIs was monitored by flow cytometry (FCM) at 48 hours. Data are presented as mean ± SD. NS, not significant; ***P < 0.001.

Having established that repetitive ADV administration induces CTL exhaustion and limits therapeutic durability (**Figure 1**), we evaluated whether engineering the virus with a CDK4/6 inhibitory function (ADV-PTD4-D3) could overcome this constraint(**Figure 2A**). To validate the functional expression and replication characteristics of the engineered ADV-PTD4-D3, the effective secretion of the target protein in infected tumor cells was first confirmed by Western blotting (**Figure 2B**). Subsequently, the infectivity and replication efficiency of the virus were systematically evaluated (**Figures 2C, D**). Colorectal cancer cell lines were infected with ADV or ADV-PTD4-D3, and cytotoxicity was assessed using CCK-8 assays (**Figure 2E**). The results indicated that both viruses exhibited comparable selective infectivity and replication kinetics within tumor cells. Crystal violet staining further confirmed that oncolytic efficacy increased in an MOI-dependent manner (**Figure 2G**). Notably, although the overall cell viability profiles were similar in CCK-8 assays, flow cytometric analysis revealed that ADV-PTD4-D3 significantly enhanced the induction of apoptosis compared to wild-type ADV (**Figure 2F**). This suggests that the CDK4/6 inhibitory payload effectively promotes immunogenic cell death mechanisms beyond simple oncolysis. Furthermore, stable expression of the EGFP reporter gene was observed in colorectal cancer cells following infection with either virus, further confirming successful viral transduction and gene expression capabilities (**Figure 2H**).

### ADV-PTD4-D3 exerts superior antitumor efficacy and induces durable immunological memory *in vivo*

**Figure 3:**
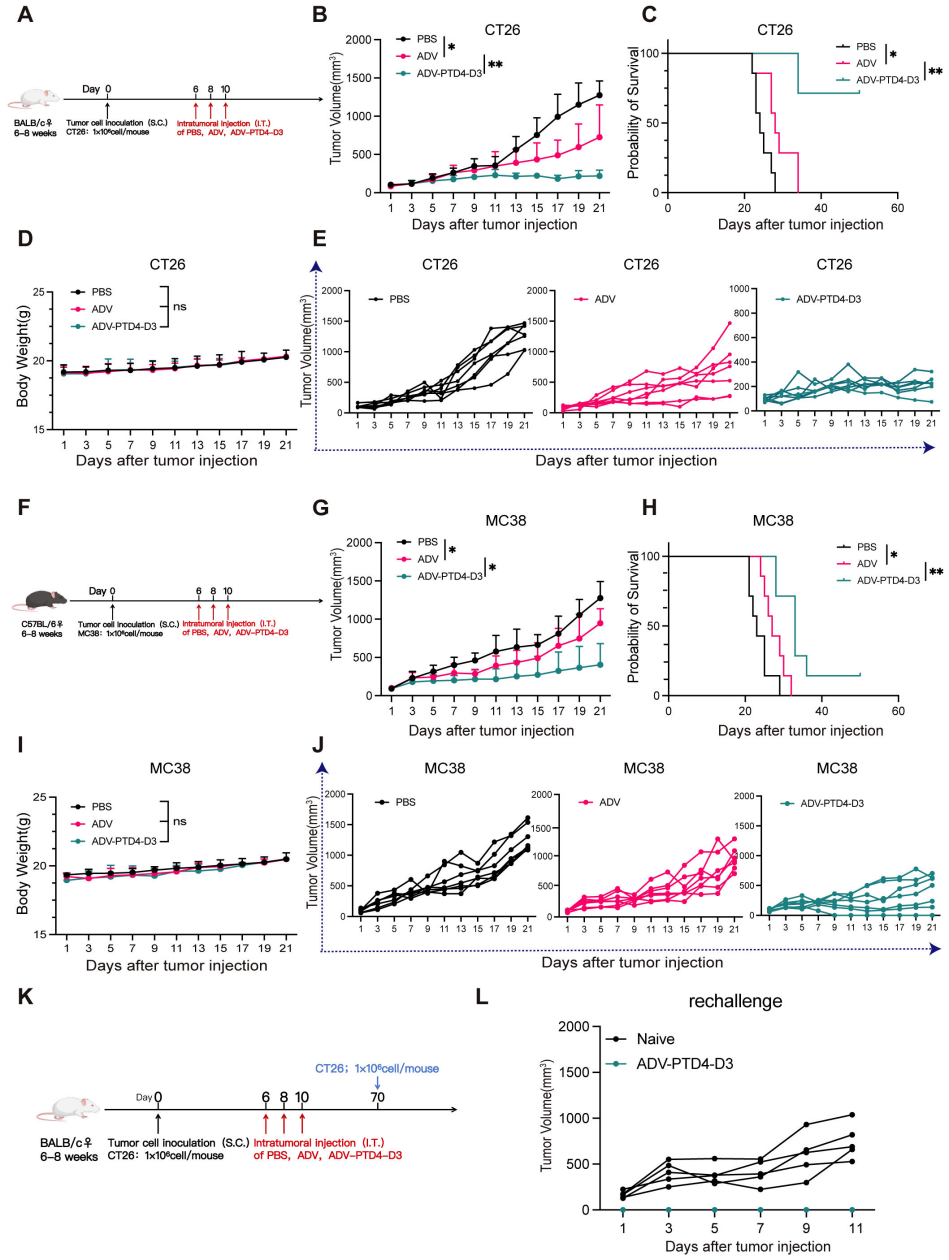
Potent therapeutic efficacy of ADV-PTD4-D3 in syngeneic colorectal cancer models. **(A)** Schematic illustration of the experimental design and treatment timeline in BALB/c mice. CT26 tumor cells were implanted subcutaneously (s.c.). Upon reaching a tumor volume of approximately 50–100 mm³, mice were randomized to receive intratumoral (i.t.) injections of PBS, ADV, or ADV-PTD4-D3 (5 × 10^8^ PFU/0.1 mL) every two days for a total of three doses. **(B–E)** Assessment of anti-tumor activity in the CT26 model (n = 7 biological replicates). Treatment commenced on day 6 post-inoculation. Panels display **(B)** average tumor growthkinetics, **(C)** Kaplan-Meier survival analysis, **(D)** body weight monitoring, and **(E)** individual tumor growth trajectories for each treatment group. Note: Residual volumes in the ADV-PTD4-D3 group represent necrotic tissue/eschar, which were confirmed to be tumor-free upon re-challenge. **(F)** Schematic of the experimental design in C57BL/6 mice using the MC38 model. The randomization and treatment protocols mirror those described in **(A)**. **(G–J)** Assessment of anti-tumor activity in the MC38 model (n = 7 biological replicates). Panels display **(G)** average tumor growth kinetics, **(H)** survival analysis, **(I)** body weight monitoring, and **(J)** individual tumor growth trajectories. **(K)** Schematic of the tumor re-challenge experiment. BALB/c mice that achieved complete regression following ADV-PTD4-D3 treatment were re-challenged s.c. with CT26 cells 30 days post-cure. Naive mice served as controls. **(L)** Tumor growth kinetics in re-challenged mice (n=5), demonstrating long-term immune memory. These 5 BALB/c mice were those that achieved complete tumor regression in the Panel **(C)** cohort following ADV-PTD4-D3 treatment. Data are presented as mean ± SD. NS, not significant; *P < 0.05, **P < 0.01.

In both CT26 (BALB/c) and MC38 (C57BL/6) syngeneic colorectal cancer models, ADV-PTD4-D3 demonstrated significantly superior tumor growth inhibition compared to wild-type ADV (**Figures 3B, G**). This enhanced efficacy translated into a substantial prolongation of survival (**Figures 3C, H**), with no adverse effects on body weight or general health (**Figures 3D, I**), indicating a favorable therapeutic window.

Remarkably, a subset of mice treated with ADV-PTD4-D3 achieved complete tumor regression (**Figures 3C, E**). In these animals, residual palpable masses, which eventually ulcerated and healed, were confirmed to consist of necrotic debris and inflammatory exudate rather than viable tumor tissue. This observation is consistent with rapid and potent oncolysis leading to durable cures.

To determine whether this profound therapeutic response engendered systemic immune protection, we performed tumor re-challenge experiments. All mice that had been cured by ADV-PTD4-D3 completely resisted re-inoculation with the same tumor cells 30 days post-regression (**Figures 3K, L**). This demonstrates the induction of potent and long-lasting antigen-specific immune memory (29, 30).

Collectively, these *in vivo* findings demonstrate that ADV-PTD4-D3 not only surpasses the antitumor efficacy of the parental oncolytic virus but also overcomes the typical limitations of virotherapy by establishing durable immunological memory. This result positions ADV-PTD4-D3 as a candidate capable of achieving sustained disease control, prompting us to investigate its underlying immunomodulatory mechanisms.

### CDK4/6 inhibition by ADV-PTD4-D3 reverses CD8+ T cell exhaustion in a CD8-dependent manner

**Figure 4:**
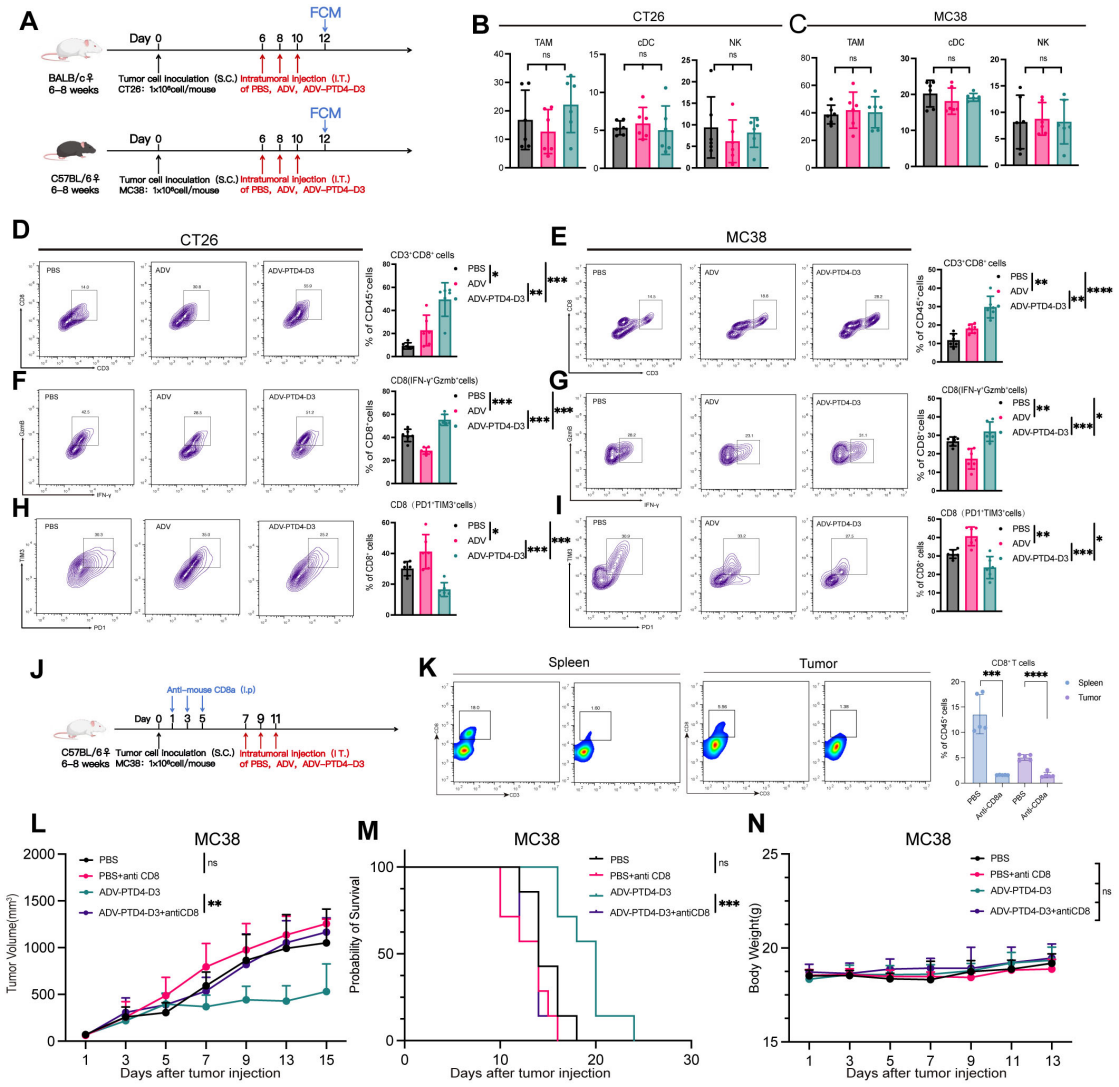
ADV-PTD4-D3 treatment enhances cytotoxic T cell infiltration and reverses functional exhaustion within the tumor microenvironment. **(A)** Schematic illustration of the experimental design and treatment schedule. Wild-type BALB/c and C57BL/6 mice bearing subcutaneous tumors (approx. 50–100 mm³) were randomized to receive intratumoral (i.t.) injections of PBS, ADV, or ADV-PTD4-D3 (5 × 10^8^ PFU) every two days for a total of three doses. Tumors were harvested for flow cytometric analysis one day after the final treatment (n = 6 biological replicates). **(B, C)** Flow cytometric quantification of innate immune cell infiltration. The percentages of tumor-associated macrophages (TAMs), natural killer (NK) cells, and dendritic cells (DCs) within the tumor microenvironment (TME) were assessed in **(B)** BALB/c and **(C)** C57BL/6 tumor models. **(D, E)** Quantification and representative flow cytometry plots of CD3+CD8+ T cell infiltration in the TME of **(D)** BALB/c and **(E)** C57BL/6 mice. **(F, G)** Assessment of effector T cell function. The frequencies of IFN-γ+Granzyme B+ cells among the CD8+ T cell population were quantified, with representative plots shown for **(F)** BALB/c and **(G)** C57BL/6 models. **(H, I)** Analysis of T cell exhaustion. The proportions of cells co-expressing inhibitory receptors (PD-1+TIM-3+) within the CD8+ T cell subset were evaluated in **(H)** BALB/c and **(I)** C57BL/6 mice. Representative plots are displayed adjacent to the quantification graphs. **(J)** Schematic of the *in vivo* CD8+ T cell depletion experiment. Tumor-bearing mice received intraperitoneal (i.p.) injections of anti-CD8α depleting antibodies concurrent with viral therapy. s.c., subcutaneous; i.t., intratumoral. **(K)** Validation of CD8+ T cell depletion efficiency. Representative flow cytometry plots and quantification of CD3+CD8+ T cell proportions in the spleen and tumor tissues following anti-CD8α treatment. **(L–N)** Impact of CD8+ T cell depletion on therapeutic efficacy. **(L)** Tumor growth kinetics, **(M)** body weight monitoring, and **(N)** Kaplan-Meier survival analysis of tumor-bearing mice across treatment groups. Data are presented as mean ± SD. NS, not significant; *P < 0.05, **P < 0.01, ***P < 0.001.

To investigate whether the superior efficacy of ADV-PTD4-D3 stemmed from modulating antitumor immunity, we profiled the tumor immune microenvironment. As expected, repetitive high-dose ADV treatment significantly increased the infiltration of CD3+CD8+ and CD3+CD4+ T cells compared to PBS control (**Figures 4D, E**, **S1A, B**). However, consistent with its suboptimal therapeutic outcome, these expanded T cell pools exhibited hallmarks of functional exhaustion: a marked increase in the co-expression of inhibitory receptors PD-1 and TIM-3 (**Figures 4H, I**), coupled with a severe reduction in IFN-γ–secreting effector CD8+ T cells (**Figures 4F, G**).

Strikingly, ADV-PTD4-D3 fundamentally reprogrammed this dysfunctional state. While it elicited a similar infiltration pattern of innate immune cells (macrophages, NK cells, DCs) as wild-type ADV (**Figures 4B, C**), the armed virus uniquely enhanced both the proportion and quality of tumor-infiltrating CD8+ T cells. ADV-PTD4-D3 not only further augmented CD8+ T cell recruitment (**Figures 4D, E**) but, crucially, preserved their effector function, as evidenced by a significant increase in IFN-γ+ cells and a concurrent decrease in the PD-1+TIM-3+ exhausted subset (**Figures 4F–I**).

To definitively establish that the rejuvenated CD8+ T cell compartment was the principal mediator of the observed therapeutic benefit, we performed *in vivo* CD8+ T cell depletion. Efficient depletion of CD8+ T cells in the periphery and tumors was confirmed (**Figures 4J, K**). In CD8-depleted mice, the potent tumor growth inhibition and survival advantage conferred by ADV-PTD4-D3 were completely abrogated (**Figures 4L–N**), underscoring the indispensable role of CD8+ T cells.

In summary, these data delineate a precise mechanism of action for our engineered virus: localized CDK4/6 inhibition via ADV-PTD4-D3 specifically rescues CD8+ T cells from activation-induced exhaustion, thereby remodeling the TME into a state conducive to tumor eradication. This CD8+ T cell-centric effect not only explains the superior efficacy over wild-type ADV but also provides a mechanistic rationale for its synergy with other T cell-directed immunotherapies.

### ADV-PTD4-D3 recruits functional CD8+ T cells via the Rb-p65-CCL5 signaling axis

The efficacy of T cell-based therapies in colorectal cancer (CRC) is often hampered by an “immune-excluded” phenotype, a state characterized by defective chemokine-mediated trafficking of lymphocytes into the tumor core (31). Activated cytotoxic T lymphocytes (CTLs) predominantly utilize CCR5 and CXCR3 receptors to follow gradients of ligands such as CCL5 and CXCL9/10/11 within the tumor microenvironment (TME) (32, 33). Notably, the abundance of these chemokines strongly correlates with CD8+ T cell infiltration density (**Supplementary Figure 1C**). We therefore hypothesized that our engineered oncolytic adenovirus could remodel this dysfunctional immune landscape by altering the tumor’s chemokine secretion profile.

**Figure 5:**
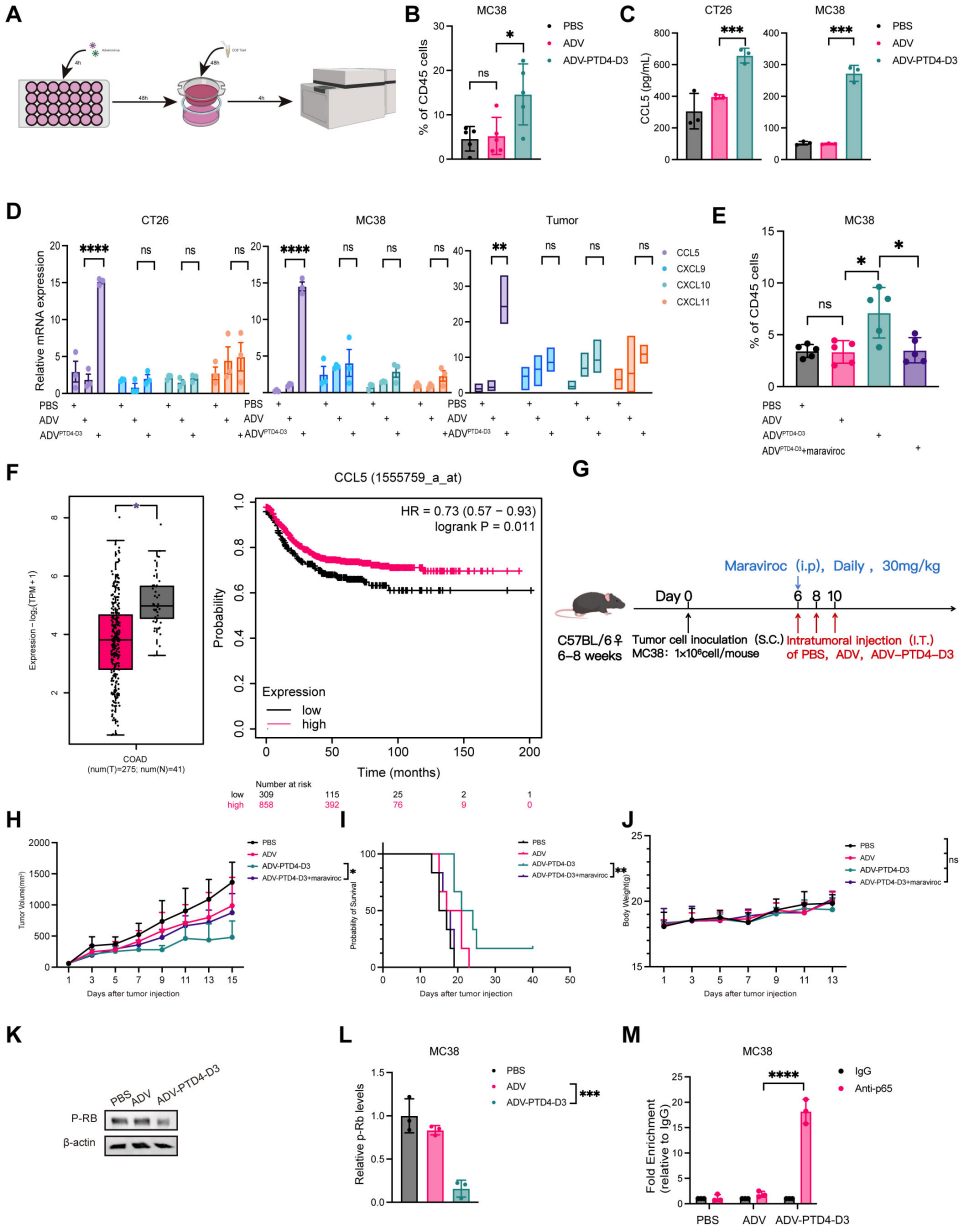
ADV-PTD4-D3 potentiates T cell-mediated immunity via the Rb-p65-CCL5 axis. **(A)** Schematic illustration of the Transwell chemotaxis assay design and timeline:Tumor cells were seeded in the lower chamber (2×10^5^ cells/well) and infected with PBS, ADV, or ADV-PTD4-D3 at an MOI of 10. After 4 hours of infection, the medium was replaced with low-serum medium (1% FBS) and cultured for an additional 48 hours to allow chemokine accumulation, followed by the addition of activated primary CD8+ T cells (2×10^5^ cells) to the upper insert to assess migration. **(B)** The migration capacity of activated CD8+ T cells towards tumor cells infected with the indicated adenoviruses was assessed using a Transwell system. Results are presented as the migration efficiency (%) (n = 5 biological replicates). **(C)** ELISA quantification of secreted CCL5 levels in the supernatants of tumor cells 48 hours after infection (MOI = 10) with the indicated adenoviruses (n = 3 biological replicates). **(D)** Transcriptional analysis of chemokine genes. Relative mRNA expression of chemokines was measured in CT26/MC38 cell lines (cultured *in vitro*) and subcutaneous tumor tissues (harvested from syngeneic mouse models) at post-treatment. ‘Tumor’ refers specifically to the excised C57BL/6 mice tumor tissues. (n = 3 biological replicates). **(E)** Impact of CCR5 blockade on T cell migration. T cell chemotaxis towards ADV-PTD4-D3-infected tumor cells was quantified in the presence or absence of a CCL5-CCR5 pathway blockade. The migration capacity of activated CD8+ T cells towards tumor cells infected with the indicated adenoviruses was assessed using a Transwell system. Results are presented as the migration efficiency (%) (n = 5 biological replicates). **(F)** Clinical significance of CCL5 in colorectal cancer. Analysis of *CCL5* mRNA expression in colon adenocarcinoma (COAD) tumor tissues (n = 275) versus normal tissues (n = 41) using the GEPIA database (*P* < 0.01). Kaplan-Meier overall survival (OS) analysis of CRC patients stratified by low (black, n = 309) versus high (red, n = 858) *CCL5* expression using the Kaplan-Meier Plotter database. Hazard Ratio (HR) and Log-rank *P* value are indicated. **(G)** Schematic of the *in vivo* mechanistic validation experiment. BALB/c mice bearing subcutaneous tumors were randomized to receive PBS, ADV, or ADV-PTD4-D3 (5 × 10^8^ PFU) combined with daily administration of Maraviroc (30 mg/kg) (n = 6 biological replicates). **(H–J)** Impact of CCL5-CCR5 blockade on therapeutic efficacy. **(H)** Tumor growth kinetics, **(I)** Kaplan-Meier survival analysis, **(J)** body weight. **(K)** Western blot analysis of phosphorylated Rb (p-Rb) levels in MC38 cells 18 hours after treatment with PBS, ADV, or ADV-PTD4-D3 (MOI = 10). **(L)** Densitometric quantification of p-Rb protein levels normalized to loading controls from three independent Western blot experiments. **(M)** Chromatin Immunoprecipitation (ChIP)-qPCR analysis. The physical occupancy of p65 at the *CCL5* promoter region was quantified in MC38 cells. Data are presented as mean ± SD. NS, not significant; *P < 0.05, **P < 0.01, ***P < 0.001, ****P < 0.0001.

To validate this hypothesis, we employed a transwell co-culture system. Tumor cells infected with ADV-PTD4-D3, but not with wild-type ADV, exhibited a significantly enhanced capacity to attract CD8+ T cells (**Figures 5A, B**). Subsequent transcriptional and protein-level screening revealed a broad upregulation of T cell-attracting chemokines upon viral infection, with CCL5 showing the most dramatic increase at both mRNA and secreted protein levels (**Figures 5C, D**). This finding holds substantial clinical relevance: analyses of public databases indicate that CCL5 expression is remarkably low in CRC tissues and inversely correlates with disease progression. Crucially, higher CCL5 transcript levels are associated with improved patient survival (**Figure 5F**), positioning its deficiency as a key contributor to poor T cell infiltration. Functional blockade of the CCR5 receptor with Maraviroc substantially abrogated the virus-induced migration of CD8+ T cells *in vitro* (**Figure 5E**). More importantly, *in vivo* co-administration of Maraviroc largely abolished the therapeutic superiority of ADV-PTD4-D3 over wild-type ADV (**Figures 5H–J**). These results unequivocally demonstrate that the enhanced T cell recruitment and anti-tumor efficacy of ADV-PTD4-D3 are critically dependent on amplifying the CCL5-CCR5 signaling axis.

Having established CCL5 as the key effector, we sought to decipher how ADV-PTD4-D3 regulates its expression. Given the virus’s engineered CDK4/6 inhibitory function and the established link between the RB1 pathway and cytokine regulation (34), we focused on this axis. Contrary to the canonical role of unphosphorylated Rb in E2F inhibition, a distinct mechanism governs its interaction with NF-κB. As demonstrated by Jin et al., hyperphosphorylated Rb (p-Rb) is the primary form that binds to and inhibits the NF-κB p65 subunit (22). Consistent with this model, treatment with ADV-PTD4-D3 effectively suppressed CDK4/6 activity, leading to a profound reduction in p-Rb levels (**Figure 5K**). This loss of p-Rb releases p65 from sequestration, allowing its nuclear translocation. To confirm that liberated p65 directly activates CCL5 transcription, we performed chromatin immunoprecipitation (ChIP) assays. The results showed a significant increase in p65 binding to a specific region of the CCL5 promoter (approximately 100–150 bp upstream of the transcription start site) following ADV-PTD4-D3 infection (**Figure 5M**).

In summary, this section defines a novel immunomodulatory pathway leveraged by our engineered oncolytic virus. ADV-PTD4-D3, via its CDK4/6 inhibitory payload, triggers Rb dephosphorylation. This, in turn, releases the NF-κB p65 subunit, which translocates to the nucleus and binds the CCL5 promoter to drive robust chemokine production. This “Rb dephosphorylation–p65 release–CCL5 transcription” axis effectively reverses the chemokine-deficient state typical of CRC, orchestrates the targeted recruitment of CD8+ T cells, and thereby establishes the essential cellular foundation for the potent tumor regression observed in our models.

### ADV-PTD4-D3 demonstrates potent efficacy in a humanized model and a favorable systemic safety profile

**Figure 6:**
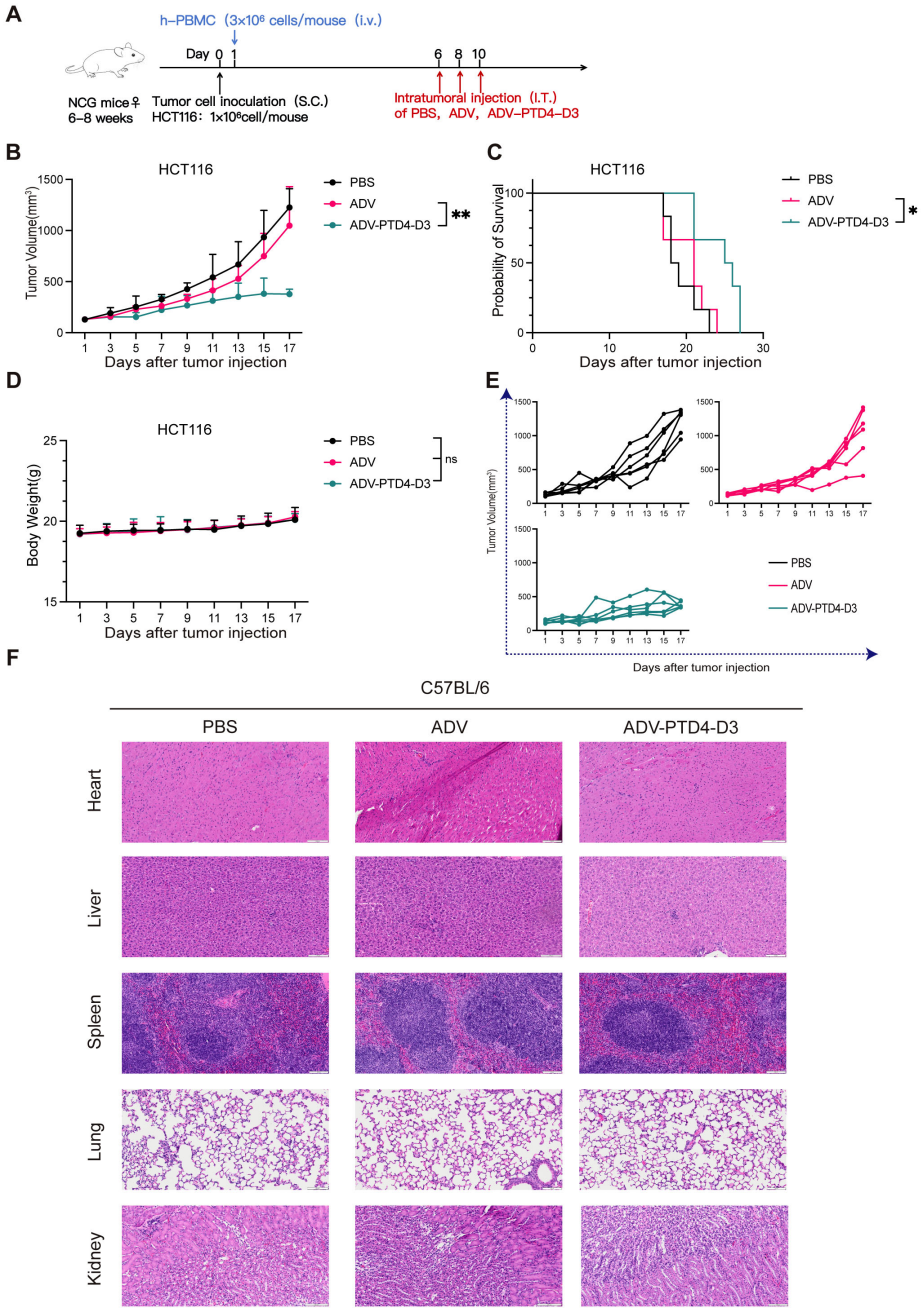
Efficacy in a humanized xenograft model and systemic safety profile of ADV-PTD4-D3. **(A)** Schematic illustration of the experimental design and treatment schedule for the humanized mouse model. Immunodeficient NCG mice were inoculated subcutaneously with tumor cells, followed by the injection of human PBMCs (3×10^6^cells/mouse) (i.v.) the next day to reconstitute the human immune system. Upon reaching a tumor volume of approximately 50–100 mm³, mice were randomized to receive intratumoral (i.t.) injections of PBS, ADV, or ADV-PTD4-D3 (5×10^8^PFU) every two days for a total of three doses. **(B–E)** Assessment of therapeutic efficacy and tolerability in the humanized model. Panels display **(B)** average tumor growth kinetics, **(C)** Kaplan-Meier survival analysis, **(D)** body weight monitoring throughout the treatment course, and **(E)** individual tumor growth trajectories for each group. **(F)** Histopathological safety assessment in immunocompetent mice. Subcutaneous MC38 tumors were established in C57BL/6 mice. Following the third administration of the recombinant virus, major organs (heart, liver, spleen, lung, and kidney) were harvested, sectioned, and subjected to Hematoxylin and Eosin (H&E) staining to evaluate potential off-target toxicity or tissue damage. Scale bars: 100 µm Data are presented as mean ± SD. NS, not significant; *P < 0.05, **P < 0.01.

To evaluate the clinical translational potential of ADV-PTD4-D3, we assessed its therapeutic efficacy in a context approximating the human immune system and its systemic safety profile in immunocompetent hosts. First, we established a humanized HCT116 colorectal cancer xenograft model (**Figure 6A**). Even under these stringent conditions, ADV-PTD4-D3 treatment resulted in tumor growth suppression significantly superior to that achieved with wild-type ADV (**Figure 6B**) and substantially prolonged host survival (**Figure 6C**), confirming its retained oncolytic and immunostimulatory potency. Critically, no significant body weight loss or signs of systemic illness were observed throughout the treatment course (**Figure 6D**), indicating excellent tolerability.

We next directly assessed the potential for off-target organ toxicity, a paramount concern for viral therapies. Histopathological examination of major organs (heart, liver, spleen, lung, kidney) from immunocompetent C57BL/6 mice after ADV-PTD4-D3 treatment revealed no abnormalities(**Figure 6F**). Bio-distribution analysis confirmed localized PTD4-D3 expression in tumors with no significant systemic leakage (**Supplementary Figure S3B**). Hematological analysis showed normal WBC and neutrophil counts, confirming systemic safety (**Supplementary Figure S3C**) These data confirm the absence of significant acute histopathological toxicity in vital organs.

In summary, ADV-PTD4-D3 exhibits a compelling therapeutic index. It maintains robust anti-tumor efficacy in a clinically relevant humanized model while demonstrating a clean systemic safety profile at the histopathological level. This “high-efficacy, low-toxicity” profile underscores its strong potential as a viable candidate for further clinical development.

### ADV-PTD4-D3 synergizes with CAR-T and anti-PD-1 therapies by remodeling the tumor immune microenvironment

To explore the translational potential of ADV-PTD4-D3, we evaluated its synergy with two established immunotherapeutic modalities: immune checkpoint blockade and adoptive cell therapy.

**Figure 7:**
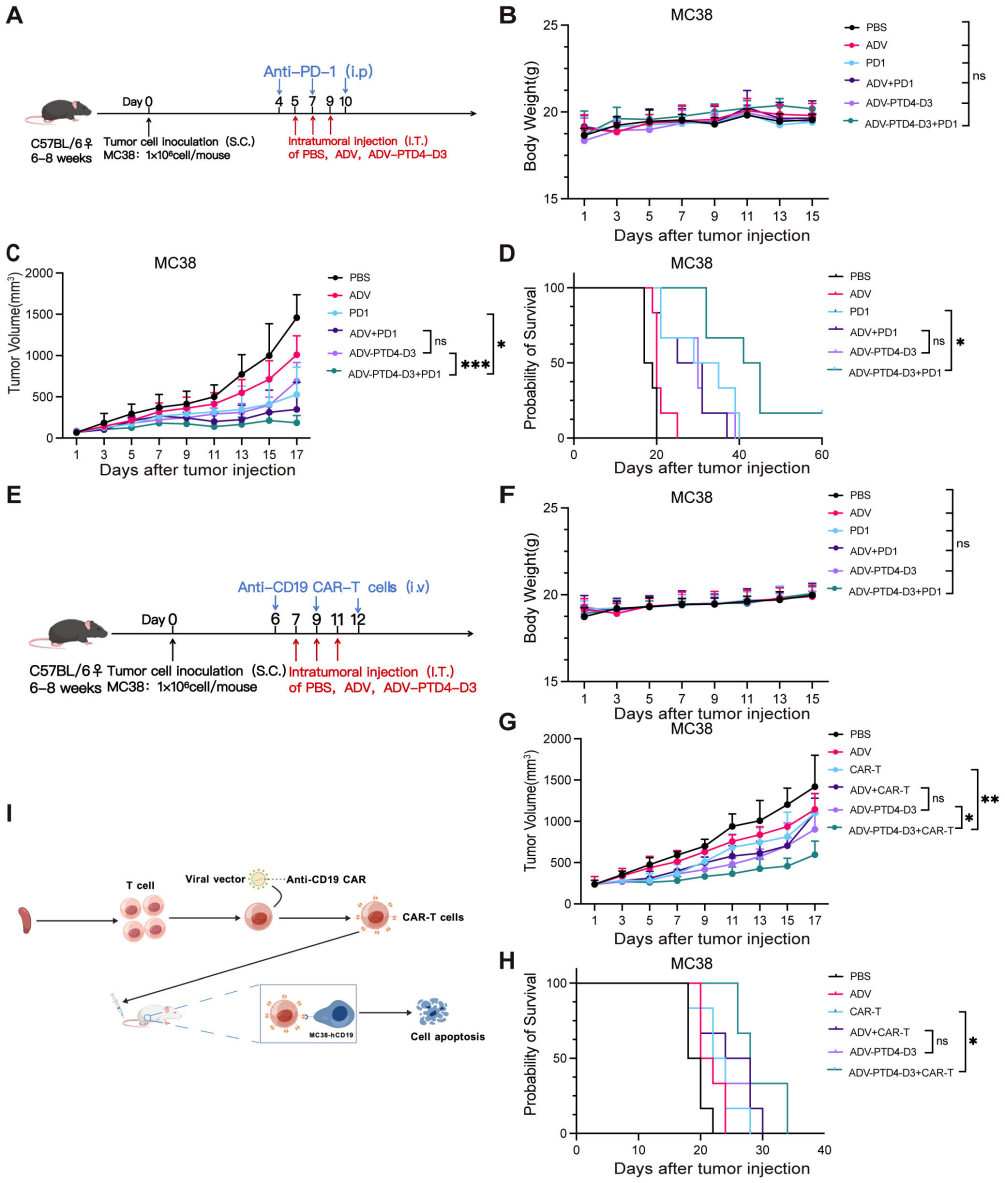
Exploration of the Translational Potential of ADV-PTD4-D3 in Combination Therapies. **(A)** Schematic illustration of the experimental design and treatment schedule for combination therapy with anti-PD-1. C57BL/6 mice bearing subcutaneous tumors (approx. 50–100 mm³) were randomized to receive intratumoral (i.t.) injections of PBS, ADV, or ADV-PTD4-D3 ( $5\times 10^{8}$PFU) every two days for three doses. Concurrent with viral therapy, mice received anti-PD-1 antibody injections(i.p.) every three days. **(B–D)** Evaluation of therapeutic efficacy in the PD-1 combination model. **(B)** Body weight monitoring, **(C)** tumor growth kinetics, and **(D)** Kaplan-Meier survival analysis of tumor-bearing mice treated with the indicated protocols. **(E)** Schematic of the combination strategy with adoptive cell therapy. One day prior to the initial viral administration, tumor-bearing mice received intravenous (i.v.) infusions of CAR-transduced T cells ( $1\times 10^{6}$ cells/mouse). CAR-T cell administration was repeated every three days for a total of three doses. **(F–H)** Assessment of anti-tumor efficacy in the CAR-T combination model. **(F)** Body weight, **(G)** tumor growth curves, and **(H)** survival rates were monitored throughout the study. **(I)** Schematic representation of CAR-T cell engineering and the therapeutic model. Murine T cells were isolated from the spleens of C57BL/6 mice and transduced with a retroviral vector encoding an anti-human CD19 CAR. Subsequently, these CAR-T cells were adoptively transferred into syngeneic mice bearing MC38-hCD19 tumors (MC38 cells engineered to stably express human CD19) to evaluate antigen-specific antitumor efficacy. Data are presented as mean ± SD. NS, not significant; *P < 0.05, **P < 0.01, ***P < 0.001.

First, we assessed the combination of ADV-PTD4-D3 and PD-1 blockade. Given that the response to PD-1 inhibitors depends on a pre-existing, tumor-reactive T cell repertoire, which is often limited in “cold” tumors, we examined whether ADV-PTD4-D3 could provide the necessary immune substrate. Our results showed that the combination enhanced the infiltration and functional profile of intratumoral CD8+ T cells, leading to a significant improvement in both tumor growth inhibition and survival rates compared to monotherapies (**Figures 7A–D**).

Next, we evaluated ADV-PTD4-D3 in combination with anti-CD19 CAR-T cell therapy. While CAR-T therapy often faces barriers in solid tumors, we hypothesized that the virus-remodelled TME could support CAR-T activity(**Supplementary Figure S3D**). *In vivo* studies demonstrated that this combination resulted in synergistic tumor growth control (**Figures 7E–H**), suggesting that ADV-PTD4-D3 can create a more permissive environment for CAR-T cell cytotoxicity in refractory models.

Altogether, ADV-PTD4-D3 remodels the immune microenvironment to overcome resistance to CAR-T and PD-1 therapies, providing a robust rationale for combination immunotherapy in solid tumors.

## Discussion

The durable efficacy of oncolytic adenoviruses in solid tumors is constrained by a paradoxical immunologic outcome: the requisite high-dose, repetitive administration induces potent CD8+ T cell infiltration but concurrently drives these cells into a state of functional exhaustion, thereby limiting therapeutic durability (**Figure 1**) (21, 35, 36), To directly intervene in this self-limiting process, we engineered ADV-PTD4-D3, an oncolytic virus armed for the intratumoral expression of a CDK4/6 inhibitory peptide. This localized delivery strategy exploits the immunomodulatory potential of CDK4/6 inhibition while circumventing the dose-limiting systemic toxicities, such as neutropenia, associated with its pharmacological administration.

A principal finding of this study is that localized CDK4/6 inhibition via ADV-PTD4-D3 fundamentally alters the fate of tumor-infiltrating CD8+ T cells. While systemic CDK4/6 inhibitors have been shown to enhance anti-tumor immunity by impeding T cell terminal differentiation and promoting memory phenotypes (37) (29), their utility is hampered by on-target toxicity in proliferative tissues. By restricting activity to the tumor microenvironment (TME), ADV-PTD4-D3 achieves precise immunomodulation: it effectively reverses the exhaustion phenotype elicited by high-dose virotherapy (**Figure 4**) and catalyzes the development of durable, antigen-specific immunological memory, as evidenced by protection against tumor re-challenge (**Figure 3**). This positions local CDK4/6 inhibition as a critical safeguard against activation-induced T cell dysfunction within the inflammatory TME.

Concurrently, ADV-PTD4-D3 addresses the deficit in effector T cell recruitment characteristic of immunologically cold tumors (38). We elucidate a novel transcriptional pathway, the Rb-p65-CCL5 axis, through which this occurs (**Figure 5**). Virus-mediated CDK4/6 inhibition dephosphorylates Rb, leading to the dissociation of the Rb-p65 complex and subsequent nuclear translocation of p65. p65 then directly binds to and transactivates the CCL5 promoter. This mechanism is distinct from previously described immunostimulatory effects of CDK4/6 inhibition, such as the induction of type III interferons via endogenous retroviral elements (10)。Given the established correlation between low CCL5 expression, poor CD8+ T cell infiltration, and adverse prognosis in colorectal cancer (33, 39). ADV-PTD4-D3 functions as a tunable intratumoral source of this key chemokine, thereby mitigating a major barrier to effective immune attack.

The dual capacity of ADV-PTD4-D3 to sustain T cell function and promote infiltration provides a robust mechanistic foundation for combination immunotherapy. For adoptive cellular therapies like CAR-T, which are limited by inefficient tumor homing and rapid exhaustion in solid tumors (39, 40), ADV-PTD4-D3 remodels the TME into a more permissive state. It provides a CCL5-mediated chemotactic gradient for recruitment and, through local CDK4/6 inhibition, helps maintain the functional potency of the engineered T cells, resulting in synergistic anti-tumor activity (**Figure 7**). Similarly, for immune checkpoint blockade, which requires a pre-existing repertoire of tumor-reactive T cells (33), ADV-PTD4-D3 acts as an essential primer. It expands and qualitatively improves the intratumoral CD8+ T cell pool, creating the necessary cellular substrate upon which PD-1/PD-L1 axis inhibition can act (**Figure 7**). This local priming approach may also offer a safety advantage by potentially reducing the severity of additive immune-related adverse events observed when systemic CDK4/6 inhibitors are combined with checkpoint blockade (41).

To contextualize the novelty of our findings, this study represents a conceptual advance over current paradigms in both CDK4/6 inhibition and oncolytic virotherapy. Regarding CDK4/6 inhibitors, existing literature predominantly focuses on systemic pharmacological administration*(*42*)*—which is frequently hindered by hematological toxicities. A research indicated that CDK4/6 inhibitor was associated with greater hematologic toxicity, especially grade 3/4 neutropenia, anemia, and thrombocytopenia (43).Our work not only pioneers the localized, virus-mediated delivery to bypass systemic toxicity but also elucidates a previously undefined immunological mechanism: the Rb-p65-CCL5 chemokine axis, directly linking cell cycle control to spatial T cell recruitment. Concurrently, in the field of oncolytic virotherapy, while it is well-established that viruses can ignite inflammation in cold tumors*(*44, 45*)*, the paradoxical induction of T cell exhaustion by repetitive viral dosing remains a unresolved bottleneck in current literature. ADV-PTD4-D3 is distinctively novel as it re-engineers the virus to self-correct this limitation, equipping the oncolytic vector with an internal mechanism to actively preserve T cell stemness and effector function against its own induced inflammatory stress.

In summary, this work re-conceptualizes the oncolytic virus from a pure cytolytic agent into a targeted delivery platform for precision immunomodulation. By engineering localized CDK4/6 inhibition, ADV-PTD4-D3 achieves a coordinated multi-faceted impact on the tumor immune microenvironment: it directly counteracts T cell exhaustion, fosters immunological memory, and augments cytotoxic lymphocyte recruitment via the novel Rb-p65-CCL5 axis. This integrated approach not only demonstrates potent standalone efficacy but also establishes a versatile combinatorial platform designed to overcome the principal barriers to current T cell-based therapies. Our findings provide a definitive preclinical proof-of-concept for a next-generation oncolytic virotherapy strategy centered on the active preservation of anti-tumor T cell function.

## Limitations and future perspectives

While our study establishes the Rb-p65-CCL5 axis as a robust mechanism for overcoming T-cell exhaustion and enhancing immunotherapy, several limitations and future research avenues warrant discussion. First, although our humanized models provide rigorous preclinical validation, they do not fully replicate the complex patterns of the human immune system in colorectal cancer. Consequently, our findings should be interpreted as a foundational concept, and future clinical trials are essential to determine the optimal dosing window and systemic tolerability in patients. Second, while our depletion assays highlight the indispensable role of CD8+ T cells, the potential synergistic contributions of other immune subsets—such as CD4+ T cells remain to be fully elucidated. Finally, while histological and physiological assessments support the localized safety profile of ADV-PTD4-D3, future pharmacokinetic studies will be instrumental in definitively mapping the peptide’s systemic biodistribution. Moving forward, we anticipate that integrating this platform with emerging therapies may further broaden its clinical utility in solid tumor immunotherapy.

## Data Availability

The original contributions presented in the study are included in the article/**Supplementary Material**. Further inquiries can be directed to the corresponding authors.

## Supplementary material

Supplementary Figure 1 Analysis of CD4+ T cell infiltration and pan-cancer correlation of CCL5. **(A)** Quantification of intratumoral CD3+CD4+ T cell infiltration in **(A)** CT26 (BALB/c) and **(B)** MC38 (C57BL/6) tumor models following the indicated treatments. Data are presented as mean ± SD (n = 6; *P < 0.05, **P < 0.01; ns, not significant). **(B)** Pan-cancer correlation analysis between CCL5 expression and CD8+ T cell infiltration. The heatmap illustrates the purity-adjusted partial Spearman’s correlation coefficients across multiple TCGA cancer cohorts, analyzed via the TIMER web server. The abundance of CD8+ T cells was estimated using various immune deconvolution algorithms (including TIMER, EPIC, MCP-counter, CIBERSORT, etc.). Red indicates a positive correlation, while blue indicates a negative correlation. Statistically significant correlations (P ≤ 0.05) are denoted by solid squares, whereas non-significant results (P>0.05) are marked with a cross (×).

Supplementary Figure 2 Gating strategy for tumor-infiltrating immune cells. **(A)** Initial gating to identify total leukocytes. Successive gating was applied to exclude debris (All Events), doublets (Singlets), and dead cells (DAPI-), followed by the identification of CD45+ leukocytes. **(B)** Identification of major myeloid and lymphoid lineages from the CD45+ population, including dendritic cells (DC; MHCII+CD11c+), tumor-associated macrophages (TAM; CD11b+F4/80+), CD4+ T cells (CD3+CD4+), and CD8+ T cells (CD3+CD8+). **(C)** Further characterization of CD8+ T cell subsets, including exhausted (PD1+TIM3+) and effector (IFN-γ+Gzmb+) populations. **(D)** Gating strategy for natural killer (NK) cells. The NK cell population (CD3-NK1.1+) was accurately identified from CD45+ cells by establishing strict gating boundaries based on the isotype control (left panel) compared to the experimental sample (right panel).

Supplementary Figure 3 ADV-PTD4-D3 induces intratumoral apoptosis and maintains systemic safety. **(A)** Western blot analysis of CAR expression in CT26-CAR and MC38-CAR cells. **(B)** Western blot analysis of HA-tagged PTD4-D3 peptide expression in tumor tissues, serum, and major organs (heart, liver, blood, tumor) harvested 48 hours after the third viral injection. **(C)** Hematological analysis of peripheral blood samples, including white blood cell count (WBC) and neutrophil count (Neu#).(n=4). **(D)** Flow cytometric analysis of human CD19 expression on MC38-CAR-hCD19 cells. **(E)** Representative immunohistochemical staining of Cleaved Caspase-3 in tumor tissues (Scale bar = 50 μm). Quantification of the Cleaved Caspase-3 positive area (%) was performed across multiple large-field images (Scale bar = 100 μm) to ensure representative sampling and is shown on the right.
