# Supplementary figures and images for: A CDK4/6 inhibitor-armed oncolytic adenovirus reverses T cell exhaustion through the Rb-p65-CCL5 pathway and potentiates the antitumor activity of anti-PD-1 or CAR-T therapy in colorectal cancer
Source: Front Immunol. 2026 Jun 15;17:1839684. doi: 10.3389/fimmu.2026.1839684 (PMC13311005; doi:10.3389/fimmu.2026.1839684)

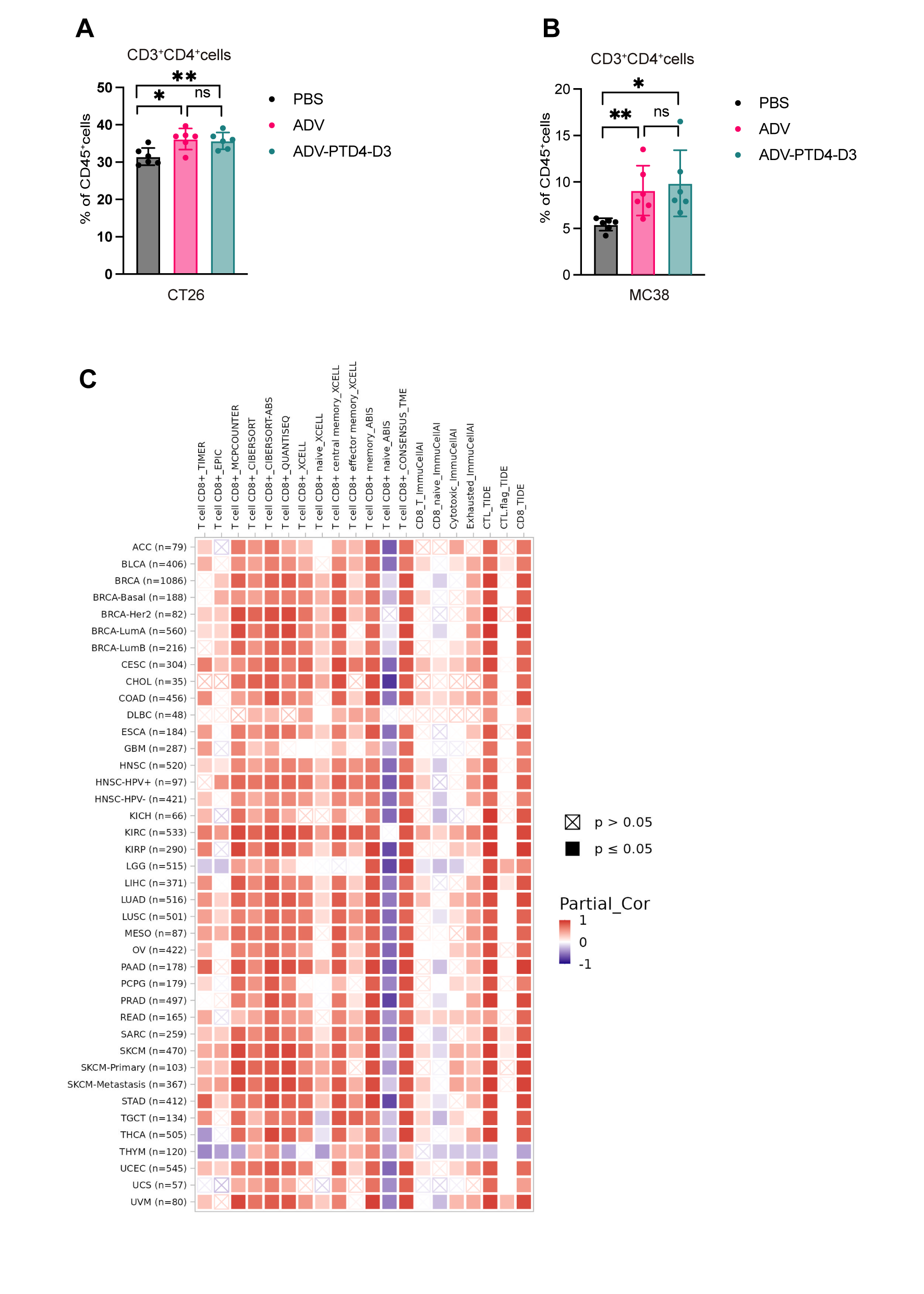

Supplement: Supplementary Figure 1 — Analysis of CD4+ T cell infiltration and pan-cancer correlation of CCL5. (A) Quantification of intratumoral CD3+CD4+ T cell infiltration in (A) CT26 (BALB/c) and (B) MC38 (C57BL/6) tumor models following the indicated treatments. Data are presented as mean ± SD (n = 6; *P < 0.05, **P < 0.01; ns, not significant). (B) Pan-cancer correlation analysis between CCL5 expression and CD8+ T cell infiltration. The heatmap illustrates the purity-adjusted partial Spearman’s correlation coefficients across multiple TCGA cancer cohorts, analyzed via the TIMER web server. The abundance of CD8+ T cells was estimated using various immune deconvolution algorithms (including TIMER, EPIC, MCP-counter, CIBERSORT, etc.). Red indicates a positive correlation, while blue indicates a negative correlation. Statistically significant correlations (P ≤ 0.05) are denoted by solid squares, whereas non-significant results (P>0.05) are marked with a cross (×). [file Image1.tif]

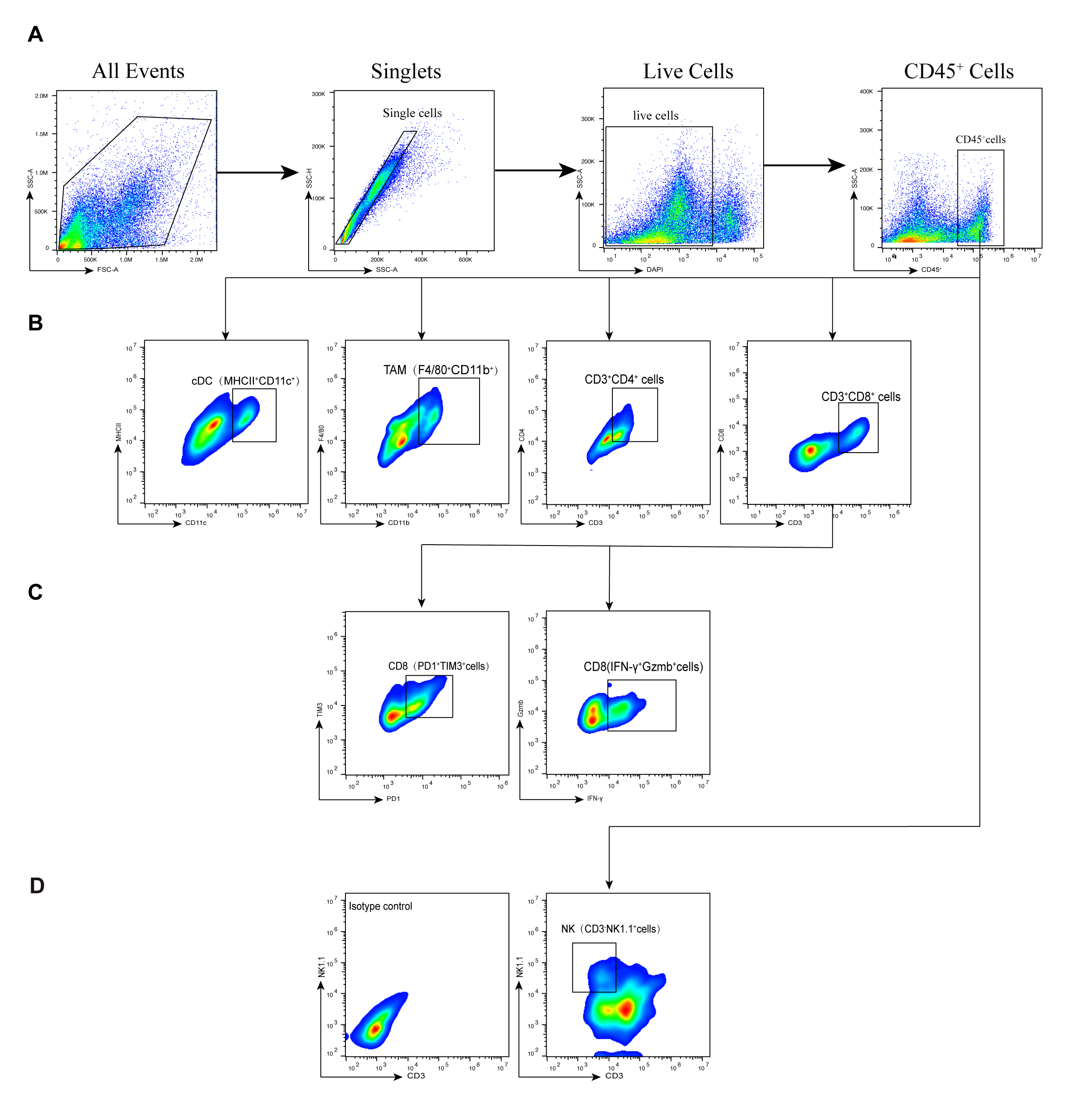

Supplement: Supplementary Figure 2 — Gating strategy for tumor-infiltrating immune cells. (A) Initial gating to identify total leukocytes. Successive gating was applied to exclude debris (All Events), doublets (Singlets), and dead cells (DAPI-), followed by the identification of CD45+ leukocytes. (B) Identification of major myeloid and lymphoid lineages from the CD45+ population, including dendritic cells (DC; MHCII+CD11c+), tumor-associated macrophages (TAM; CD11b+F4/80+), CD4+ T cells (CD3+CD4+), and CD8+ T cells (CD3+CD8+). (C) Further characterization of CD8+ T cell subsets, including exhausted (PD1+TIM3+) and effector (IFN-γ+Gzmb+) populations. (D) Gating strategy for natural killer (NK) cells. The NK cell population (CD3-NK1.1+) was accurately identified from CD45+ cells by establishing strict gating boundaries based on the isotype control (left panel) compared to the experimental sample (right panel). [file Image2.tif]

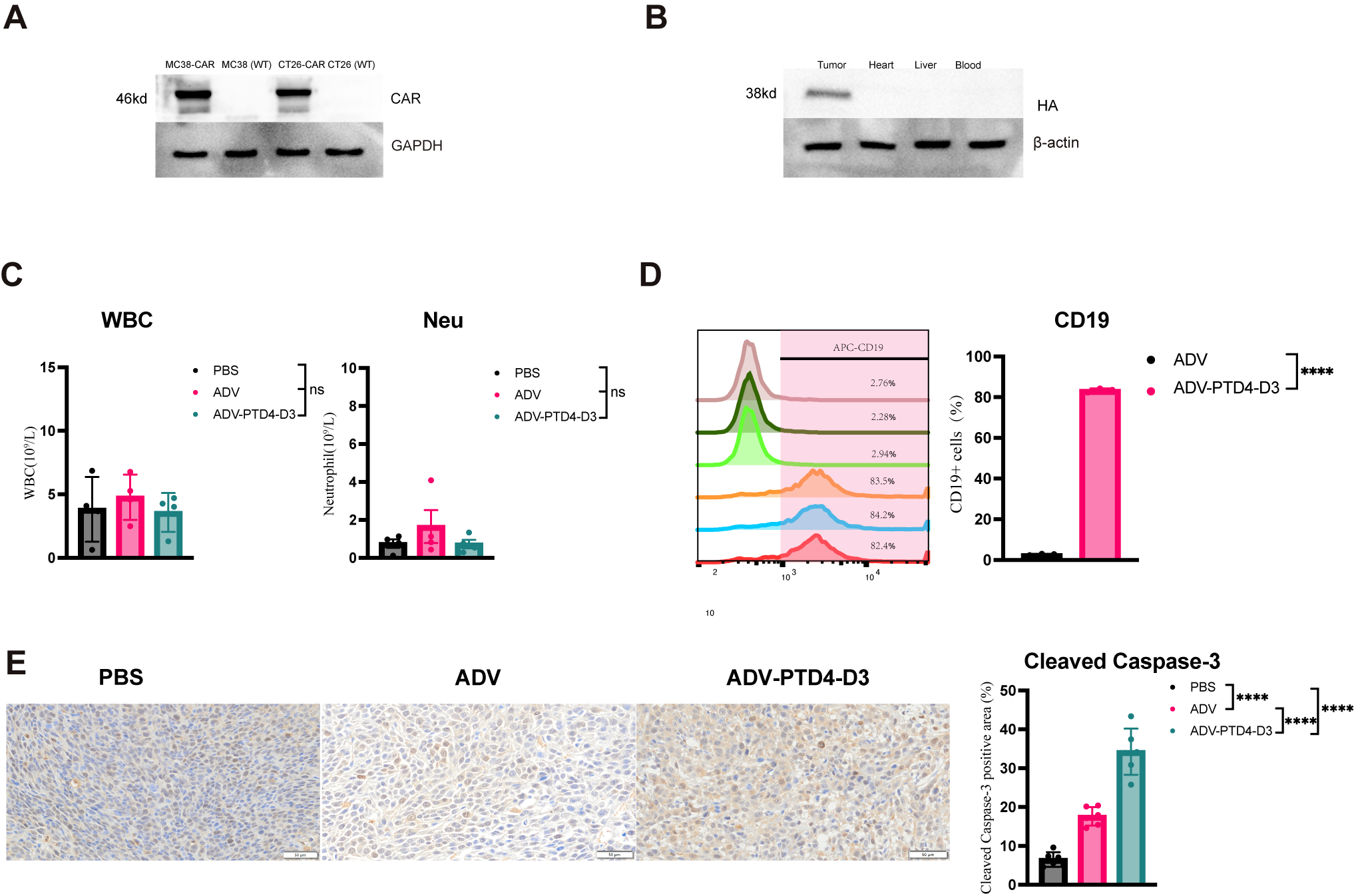

Supplement: Supplementary Figure 3 — ADV-PTD4-D3 induces intratumoral apoptosis and maintains systemic safety. (A) Western blot analysis of CAR expression in CT26-CAR and MC38-CAR cells. (B) Western blot analysis of HA-tagged PTD4-D3 peptide expression in tumor tissues, serum, and major organs (heart, liver, blood, tumor) harvested 48 hours after the third viral injection. (C) Hematological analysis of peripheral blood samples, including white blood cell count (WBC) and neutrophil count (Neu#).(n=4). (D) Flow cytometric analysis of human CD19 expression on MC38-CAR-hCD19 cells. (E) Representative immunohistochemical staining of Cleaved Caspase-3 in tumor tissues (Scale bar = 50 μm). Quantification of the Cleaved Caspase-3 positive area (%) was performed across multiple large-field images (Scale bar = 100 μm) to ensure representative sampling and is shown on the right. [file Image3.tif]
