# Supplementary material for: A CDK4/6 inhibitor-armed oncolytic adenovirus reverses T cell exhaustion through the Rb-p65-CCL5 pathway and potentiates the antitumor activity of anti-PD-1 or CAR-T therapy in colorectal cancer
Source: Front Immunol. 2026 Jun 15;17:1839684. doi: 10.3389/fimmu.2026.1839684 (PMC13311005; doi:10.3389/fimmu.2026.1839684)
Supplement: Supplementary file 4 [file Table1.pdf]

Supplementary Table S1. Primer sequences.

Target name	Forward primer (5’-3’)	Reverse primer (5’-3’)
GAPDH	ATCAAGAAGGTGGTGAAGCA	AGACAACCTGGTCCTCAGTGT
Hexon	ACCGTGAGGATACTGCGTAC	TTGCTCGTCTACTTCGTCTT
CCL5	GCTGCTTTGCCTACCTCTCC	TCGAGTGACAAACACGACTGC
CXCL9	TCCTTTTGGGCATCATCTTCC	TTTGTAGTGGATCGTGCCTCG
CXCL10	CCAAGTGCTGCCGTCATTTTC	GGCTCGCAGGGATGATTTCAA
CXCL11	GGCTTCCTTATGTTCAAACAGGG	GCCGTTACTCGGGTAAATTACA

Supplementary Table S2 Peptide sequences.

Peptide	Sequence
PTD4—D3	YARAAARQARA-GAVSPRQLRDWEVLVLGK
IgG—Fc	VPRDCGCKPCICTVPEVSSVFIFPPKPKDVLTITLTPKVTCVVVDISKDDPEVQFSWFVDDVE VHTAQTQPREEQFNSTFRSVSELPIMHQDWLNGKEFKCRVNSAAFPAPIEKTISKTKGRPK APQVYTIPPPKEQMAKDKVSLTCMITDFFPEDITVEWQWNGQPAENYKNTQPIMNTNGSY FVYSKLNVQKSNWEAGNTFTCSVLHEGLHNHHTEKSLSHSPGK
